# Toosendanin activates caspase-1 and induces maturation of IL-1β to inhibit type 2 porcine reproductive and respiratory syndrome virus replication via an IFI16-dependent pathway

**DOI:** 10.1186/s13567-022-01077-2

**Published:** 2022-07-29

**Authors:** Mingxin Zhang, Chunni Lu, Lizhan Su, Feixiang Long, Xia Yang, Xiaofeng Guo, Gaopeng Song, Tongqing An, Weisan Chen, Jianxin Chen

**Affiliations:** 1 Guangdong Provincial Key Laboratory of Veterinary Pharmaceutics Development and Safety Evaluation, College of Veterinary Medicine, South China Agricultural University, Guangzhou, 510642 China; 2 Centre for Inflammatory Diseases, Department of Medicine, Monash Medical Centre, Monash University, Monash University, Clayton, VIC 3168 Australia; 3 College of Materials and Energy, South China Agricultural University, Guangzhou, 510642 China; 4 State Key Laboratory of Veterinary Biotechnology, Harbin Veterinary Research Institute, Chinese Academy of Agricultural Sciences, Harbin, 150069 China; 5 Department of Biochemistry and Genetics, La Trobe Institute for Molecular Science, La Trobe University, Melbourne, VIC 3086 Australia

**Keywords:** Porcine reproductive and respiratory syndrome virus (PRRSV), Toosendanin (TSN), inhibitory activity, gamma-interferon-inducible protein 16 (IFI16), caspase-1; Interleukin-1β (IL-1β)

## Abstract

Porcine reproductive and respiratory syndrome virus (PRRSV) is a prevalent and endemic swine pathogen which causes significant economic losses in the global swine industry. Multiple vaccines have been developed to prevent PRRSV infection. However, they provide limited protection. Moreover, no effective therapeutic drugs are yet available. Therefore, there is an urgent need to develop novel antiviral strategies to prevent PRRSV infection and transmission. Here we report that Toosendanin (TSN), a tetracyclic triterpene found in the bark or fruits of *Melia toosendan* Sieb. et Zucc., strongly suppressed type 2 PRRSV replication in vitro in Marc-145 cells and ex vivo in primary porcine alveolar macrophages (PAMs) at sub-micromolar concentrations. The results of transcriptomics revealed that TSN up-regulated the expression of IFI16 in Marc-145 cells. Furthermore, we found that IFI16 silencing enhanced the replication of PRRSV in Marc-145 cells and that the anti-PRRSV activity of TSN was dampened by IFI16 silencing, suggesting that the inhibition of TSN against PRRSV replication is IFI16-dependent. In addition, we showed that TSN activated caspase-1 and induced maturation of IL-1β in an IFI16-dependent pathway. To verify the role of IL-1β in PRRSV infection, we analyzed the effect of exogenous rmIL-1β on PRRSV replication, and the results showed that exogenous IL-1β significantly inhibited PRRSV replication in Marc-145 cells and PAMs in a dose-dependent manner. Altogether, our findings indicate that TSN significantly inhibits PRRSV replication at very low concentrations (EC_50_: 0.16–0.20 μM) and may provide opportunities for developing novel anti-PRRSV agents.

## Introduction

Porcine reproductive and respiratory syndrome (PRRS) has been a great concern to pig industry globally since it was discovered in 1987 in the United States [1]. It has been estimated that PRRS infection leads to at least $600 million losses annually to the US swine industry [2, 3]. PRRS virus (PRRSV), the etiological agent of PRRS, is an enveloped virus containing a single-stranded, positive-sense RNA genome of 15.4 kb in length. It belongs to the family of *Arteriviridae* in the order *Nidovirales* [4]. Currently, the prevention of PRRS infection mainly depends on vaccination, including the use of inactivated and attenuated live vaccines. However, the vaccines usually provide limited protection against PRRS infection [5, 6] due to high mutation rate of PRRSV and that the circulation of multiple subtypes [7]. Some compounds may act as potential anti-PRRSV agents, such as artesunate [8], glycyrrhizin [9], xanthohumol [10], platycodin D [11], (−)-epigallocatechin-3-gallate [12], and flavaspidic acid AB [13]. Nevertheless, no effective drugs are commercially available for the treatment of PRRSV infection.

Innate immune defenses are essential for the control of virus infection [14]. Inflammation is a tightly controlled process induced by the innate immune system in response to virus infection and is responsible for virus clearance and homeostasis restoration [15]. It is triggered by the sensing of Pathogen-Associated Molecular Patterns (PAMPs) by Pattern Recognition Receptors (PRRs) [15, 16]. PRRs include the cytosolic receptors such as the membrane-anchored Toll-like receptors (TLRs), nucleotide-binding and oligomerization domain (NOD)-like receptors (NLRs), retinoic acid-inducible gene I (RIG-I)-like receptors (RLRs) and cyclic GMP-AMP synthase (cGAS) [17]. It is well known that the gamma-interferon-inducible protein 16 (IFI16) acts as a nuclear sensor and a direct restriction factor of a variety of DNA viruses [18–20]. Existing evidence shows that IFI16 also directly senses viral RNA and enhances RIG-I transcription and activation to restrict RNA virus infection [21, 22]. Chang et al. showed that IFI16 plays a key role in regulating the type I IFN response to PRRSV, and overexpression of IFI16 inhibits PRRSV replication in Marc-145 cells [23]. These findings indicate that upregulation of IFI16 can potentially combat PRRSV infection. However, it remains unclear whether IFI16 affects PRRSV infection in primary porcine alveolar macrophages (PAMs), which are a major target of PRRSV in pigs.

The proinflammatory cytokine IL-1β, mainly produced by macrophages, epithelial cells and neutrophils, plays important roles in host defense against virus infection [24, 25]. The caspase-1 activation itself is regulated by a multi-protein complex known as the inflammasome. The inflammasome is a caspase-1-activating molecular platform formed by interaction of three proteins, 1) a ‘‘sensor protein’’ recognizing the trigger, 2) an adaptor molecule known as the apoptosis associated speck-like protein containing CARD (ASC), and 3) procaspase-1. This platform provides the molecular scaffolds required for the proteolytic processing of inactive procaspase-1 in to active caspase-1 [26, 27]. IL-1β was found to have potent antiviral effects against the infection of a variety of viruses, including hepatitis B virus (HBV) [28], influenza A virus (IAV) [29] and African swine fever virus (ASFV) [30]. However, the role of the IFI16 inflammasome and its downstream factor IL-1β in PRRSV infection remain unclear.

Toosendanin (TSN) is a tetracyclic triterpene extracted from the bark and fruits of *Melia toosendan* Sieb. et Zucc. It has been used as agricultural insecticide for decades and was ever used as digestive tract parasiticide in China [31–33]. In 2004, TSN was demonstrated to have a marked anti-botulismic effect in vivo and in vitro [31]. After that, TSN was reported to possess anticancer activities by inducing apoptosis in various cancer cells [34–36]. In recent years, TSN was reported to have antiviral activities against IAV [37], hepatitis C virus (HCV) [38], severe fever with thrombocytopenia syndrome virus (SFTSV) and SARS-CoV-2 [39]. However, the molecular mechanism of action for TSN against these viral infections remains poorly understood.

In the present study, we show that TSN potently inhibits PRRSV infection at sub-micromolar concentrations in a dose-dependent manner in Marc-145 cells and in PAMs. Furthermore, we demonstrate that inhibition of PRRSV infection by TSN is associated with the up-regulation of IFI16 and the activation of its downstream caspase-1/IL-1β signaling pathways. To our knowledge, this is the first report of the mechanisms responsible for TSN anti-PPRSV activities. Our work may provide valuable insight into the application of TSN as a novel antiviral against PRRS infection.

## Materials and methods

### Cell lines and viruses

Marc-145 cells, a PRRSV-permissive cell line derived from African green monkey kidney cell line MA-104 were obtained from the American Type Culture Collection (ATCC) and grown in Dulbecco’s minimum essential media (DMEM, Gibco, UT, USA) supplemented with 10% fetal bovine serum (FBS, Biological Industries, Kibbutz Beit Haemek, Israel) and 100 IU/mL of penicillin and 100 g/mL streptomycin at 37 °C with 5% CO_2_.

Porcine alveolar macrophages (PAMs) were obtained from the lungs of 4- to 6-week-old PRRSV-negative Large-White piglets (Xinli Pig Farm, Wuzhou, China) by lung lavage according to a previously described method [40]. Briefly, the lungs were washed three times with pre-cooled phosphate buffered saline (PBS) solution containing penicillin (300 IU/mL) and streptomycin (300 g/mL). Cells were centrifuged at 800 g for 10 min, resuspended in RPMI 1640 supplemented with 10% FBS and 100 IU/mL of penicillin and 100 g/mL streptomycin at 1 × 10^6^ cells/mL in 6-well plate, and then incubated at 37 °C for 2 h.

Three type 2 PRRSV strains including classical CH-1a and VR-2332 and the highly pathogenic PRRSV NADC30-like HNhx strain (GenBank no. KX766379) [41] were propagated in Marc-145 cells in DMEM containing 3% FBS (essential media). Virus preparations were titrated and stored at −80 ℃. Virus titer was determined using a microtitration infectivity assay [42].

### Preparation of toosendanin and chemicals

Toosendanin (TSN) and Platycodin D (PD) were purchased from Chengdu Pu fei De Biotech Co., Ltd. (Chengdu, China), with a purity of 99.3%. Ribavirin (Rib), a broad-spectrum antiviral was purchased from Star Lake Bioscience Co., Ltd. (Zhaoqing, China). It was dissolved in dimethyl sulfoxide (DMSO, Sigma-Aldrich, MA, USA) and diluted with essential medium before use. The final concentration of DMSO in the culture media was less than 0.4%. Recombinant porcine Interleukin-1β (rmIL-1β) was purchased from Beyotime Biotechnology (Shanghai, China). It was reconstituted in sterile distilled water to make up a 0.1–1.0 mg/mL stock solutions. The rmIL-1β stock solutions were aliquoted and stored at −20 ℃.

### Cytotoxicity assay

The cytotoxicity of TSN was evaluated using MTT assay [13]. Briefly, Marc-145 cells (5 × 10^4^ cells/well) and PAMs (2 × 10^5^ cells/well) were seeded in 96-well plates and grown at 37 °C for 24 h and 6 h, respectively. The media were then replaced with fresh media containing serial dilutions of TSN, followed by incubation for 48 h. After removal of the culture media, the cells were incubated with 100 μL 3-(4,5-dimethylthiozol-2-yl)-3, 5-dipheryl tetrazolium bromide (MTT; Sigma-Aldrich) solution (0.5 mg/mL in PBS) at 37 °C for 4 h. The MTT was then removed, and 150 μL of DMSO was added into each well to dissolve the formazan crystals for 10 min at 37 °C. Cell viability was measured by absorbance at 490 nm with a microplate reader (Thermo fisher scientific, MA, USA) and expressed as a percentage of the control level. The mean optical density (OD) values from six wells per treatment were used as the cell viability index. The 50% cytotoxic concentration (CC_50_) was analyzed by GraphPad Prism 5.0 (GraphPad Software, San Diego, CA, USA).

### Viral titer titration

Viral titer was established with the endpoint dilution assay described as before [42]. Briefly, viral preparations were serially diluted in essential media in tenfold increments, and 100 µL of each diluted preparation was inoculated in quadruplicate to confluent monolayers of Marc-145 cells in 96-well plates, followed by incubation for 2 h at 37 °C. The inoculum was then aspirated out from each well, and the cell monolayers were replenished with fresh essential media and cultured for an additional 72 h to monitor daily the cytopathic effects (CPE). Virus titer was calculated based on the amount of CPE and expressed as a 50% tissue culture infective dose (TCID_50_)/mL.

### Indirect immunofluorescence assay (IFA)

For immunostaining, the PRRSV-infected or uninfected cells were fixed with 4% paraformaldehyde for 10 min, permeabilized with 0.25% Triton X-100 for 10 min at room temperature (RT), blocked with 1% bovine serum albumin (BSA) for 60 min at RT and then incubated with a mouse monoclonal antibody against the N-protein of PRRSV (clone 4A5, 1:400 dilution, MEDIAN Diagnostics, Korea) at 4 ℃ overnight. After three washes with PBS, the cells were incubated for 1 h at RT with a goat anti-mouse secondary antibody conjugated with Alexa Fluor 568 (red) (Cell Signaling Technology, MA, USA) at 1:1000 dilution. Nuclei were counterstained using 50 μL of 4,6-diamidino-2-phenylindole (DAPI, 300 nM; Sigma-Aldrich) (blue). Immunofluorescence was captured using the Leica DMI 4000B fluorescence microscope (Leica, Wetzlar, Germany). Blue and red fluorescence spots were counted as the total and PRRSV-infected cell number, respectively, in every IFA image. The percentage of infected cell counts among total cell counts was considered as the infection rate. Relative infected-cell percentage was determined by the ratio of the infection rate in TSN-treated groups to that in DMSO-treated control. The EC_50_ value (the concentration required to protect 50% cells from PRRSV infection) was determined by plotting the relative infected-cell percentage as a function of compound concentration and calculated with the GraphPad Prism 5.0 software.

### Real-Time quantitative reverse-transcription PCR (qRT-PCR)

Total RNA was extracted using the total RNA rapid extraction kit (Fastagen, Shanghai, China) following the manufacturer’s instructions. RNA was reverse-transcribed into first-strand cDNA using a reverse transcription kit (Genstar, Beijing, China). PCR amplification was performed on 1 μL of reverse-transcribed product with primers designed against PRRSV-NSP9, IFI16 and GAPDH (glyceraldehyde-3-phosphate dehydrogenase, used as endogenous control). The primers used for PCR amplification are listed in Table 1 [13, 23]. Real-time PCR was performed using 2 RealStar Green Power Mixture (containing SYBR Green I Dye) (Genstar, Beijing, China) on the CFX96 Real-time PCR system (Bio-Rad, CA, USA). Relative mRNA expression was calculated by 2^−ΔΔCT^ method using DMSO-treated infected cells or DMSO-treated mock-infected cells as reference samples for determining PRRSV-NSP9 and IFI16 gene expression, respectively [43, 44].

**Table 1:** **Real-time PCR primer sequences**

Name^a^	Sequences (5' to 3')
NSP9-F NSP9-R GAPDH-F GAPDH-R IFI16-F IFI16-R	5'-CTAAGAGAGGTGGCCTGTCG-3' 5'-GAGACTCGGCATACAGCACA-3' 5'-GCAAAGACTGAACCCACTAATTT-3' 5'-TTGCCTCTGTTGTTACTTGGAGAT-3' 5'-CGCGGATCCATGGAAAAAAAATACAAGAACATTG-3' 5'-CCGCTCGAGTTAGAAGAAAAAGCCTGGTGAAGTT-3'

^a^F: forward primer R: reverse primer

### Western blot analysis

PRRSV-infected or uninfected Marc-145 cells treated with TSN were lysed in RIPA lysis buffer containing 1 mM phenylmethylsulfonylfluoride (Beyotime, Shanghai, China) at 4 ℃. The supernatant was harvested after centrifugation (15 000 rpm for 30 min at 4 ℃) and the total protein for each sample measured using the BCA protein assay kit (Beyotime, China). 10 μg of total protein per sample was electrophoresed onto 12% SDS-PAGE gels and transferred onto polyvinylidene-fluoride (PVDF) membranes (Millipore, MA, USA). After blocking, membranes were incubated with a mouse anti-PRRSV N-protein monoclonal antibody (clone 4A5, MEDIAN Diagnostics, Chuncheon, Korea), IFI16 rabbit polyclonal Antibody (Beyotime, Shanghai, China), Gasdermin D rabbit monoclonal antibody (Cell Signaling Technology, MA, USA), Cleaved Gasdermin D rabbit monoclonal antibody (Cell Signaling Technology, MA, USA), pro-caspase-1 rabbit monoclonal antibody (Cell Signaling Technology, MA, USA), caspase-1 rabbit monoclonal antibody (Cell Signaling Technology, MA, USA), IL-1β rabbit monoclonal antibody (Cell Signaling Technology, MA, USA) or a mouse anti-GAPDH monoclonal antibody (GoodHere, Hangzhou, China) at 1:1000 dilution at 4 ℃ overnight. HRP conjugated goat anti-mouse or anti-rabbit IgG (H–L) (1:3000; Beyotime, Shanghai, China) was used as the secondary antibody for 1 h incubation at RT. The Western blot results were visualized using the chemiluminescence technology.

### Antiviral activity assay

The in vitro antiviral activity assay was performed to examine the capacity of TSN to inhibit PRRSV replication. Marc-145 cells or PAMs in 96-well plates were infected with PRRSV at a multiplicity of infection (MOI) of 0.05 for Marc-145 cells and 0.5 for PAMs in essential medium for 2 h at 37 ℃. The culture supernatants were then removed and fresh DMEM containing different concentrations of each compound were added into each well. Cells and supernatants were then collected at the indicated time points post infection, and their virus titer was determined by the endpoint dilution assay and viral RNA level by qRT-PCR. Cells were subjected to PRRSV-infected cell counting by IFA, and their viral NP level was determined by Western blotting.

### Direct PRRSV-TSN interaction

To investigate whether TSN directly interact with the virus, 100 μL of PRRSV (0.5 MOI) was mixed with various TSN concentrations in essential medium (0.9 mL) and incubated for 1 h at 37 ℃. Then, PRRSV and TSN were separated by ultrafiltration centrifugation. PRRSV trapped in the ultrafiltration tube were washed three times with essential medium to remove residual TSN, and were then resuspended in essential medium to infect Marc-145 cells grown in 12-well plates for 2 h. After three washes with PBS, the cells were cultured in fresh medium for an additional 48 h at 37 ℃ and then subjected to viral mRNA and N protein using qRT-PCR and IFA.

### TSN pretreatment on Marc-145 cells

To investigate whether TSN inhibits PRRSV replication through altering the susceptibility of host cells, Marc-145 cells were pretreated with TSN in essential medium for 2 h at 37 ℃. After three washes with PBS, cells were infected with PRRSV (0.05 MOI) for 2 h and collected at 48 h post-infection (hpi) for qRT-PCR and IFA.

### RNA collection, preparation, transcriptome sequencing, and functional analysis

Marc-145 were grown in 6-well plates were infected with PRRSV (0.05 MOI) in essential medium for 2 h at 37 ℃. Supernatants were removed and fresh DMEM containing 1.6 μM of TSN or DMSO were added and incubated for 2 h and then the cells were collected for transcriptomic analysis. Transcriptome sequencing was performed in the Novogene Bioinformatics Institute (Novogene, Beijing, China). Briefly, mRNA was purified from total RNA using poly-T oligo-attached magnetic beads. Fragmentation was carried out using divalent cations under elevated temperature in First Strand Synthesis Reaction Buffer (5X). First strand cDNA was synthesized using random hexamer primer and M-MuLV Reverse Transcriptase (RNase H-). Second strand cDNA synthesis was subsequently performed using DNA Polymerase I and RNase H. Remaining overhangs were converted into blunt ends via exonuclease/polymerase activities. After adenylation of 3 ends of DNA fragments, Adaptor with hairpin loop structure were ligated to prepare for hybridization. In order to select cDNA fragments of preferentially 370–420 bp in length, the library fragments were purified with AMPure XP system (Beckman Coulter, Beverly, USA). Then PCR was performed with Phusion High-Fidelity DNA polymerase, Universal PCR primers and Index (X) Primer. At last, PCR products were purified (AMPure XP system) and library quality was assessed on the Agilent Bioanalyzer 2100 system. The clustering of the index-coded samples was performed on a cBot Cluster Generation System using TruSeq PE Cluster Kit v3-cBot-HS (Illumia) according to the manufacturer instructions. After cluster generation, the library preparations were sequenced on an Illumina Novaseq platform and 150 bp paired-end reads were generated. Feature Counts v1.5.0-p3 was used to count the reads numbers mapped to each gene. The fragments per kilo base per million (FPKM) of each gene was calculated based on the length of the gene and reads count mapped to this gene. Different expression analysis of two conditions/groups (two biological replicates per condition) was performed using the DESeq2 R package (1.20.0). DESeq2 provides statistical routines for determining differential expression in digital gene expression data using a model based on the negative binomial distribution. The resulting *P*-values were adjusted and the Benja mini and Hochberg approach was used to decrease the false discovery rate. Genes with an adjusted *P*-value < 0.05 found by DESeq2 were assigned as differentially expressed. We used cluster Profiler R package to test the statistical enrichment of differential expression genes in Kyoto Encyclopedia of Genes and Genomes (KEGG) pathways.

### Gene silencing with siRNA

The specific siRNAs targeting IFI16 (si-IFI16) and the control siRNA (siNC) were synthesized by Sangon Biotech. The siRNA sequences are as follows: siIFI16, 5′-CCAAGCAGCAGUUGCUUAATT-3′; the nontargeting control siRNA (siNC), 5′-UUCUCCGAACGUGUCACGUTT-3′ [23]. Marc-145 cells were transfected with 50 nM siRNA using 5 µL of Lipofectamine™ 3000 Transfection Reagent (Thermo Scientific, CA, USA). 24 h post-transfection (hpt), the transfected cells were infected with PRRSV and treated with TSN (0.4 μM). Supernatants and lysates of the cells were then collected at 24 hpi for further analysis.

### Determination of GSDMD-N, Caspase-1 and IL-1β levels

Marc-145 cells or PAMs in 6-well plates were infected with PRRSV (0.05 MOI for Marc-145 cells and 0.5 MOI for PAMs) in essential medium for 2 h at 37 ℃. The culture supernatants were then removed and fresh DMEM containing different TSN concentrations were added into each well. Cells were cultured in fresh medium for an additional 24 h at 37 ℃ and then subjected to GSDMD-N, Caspase-1 and IL-1β protein using Western blotting.

### Statistical analysis

All experiments were performed at least three times. The results were presented as mean standard deviation (SD). Statistical significance was determined by the Student’s *t* test when only two groups were compared or by one-way analysis of variance (ANOVA) when more than two groups were compared. *p < 0.05, **p < 0.01, and ***p < 0.001 were considered to be statistically significant at different levels.

## Results

### TSN inhibits PRRSV infection in Marc-145 cells

**Figure 1:**
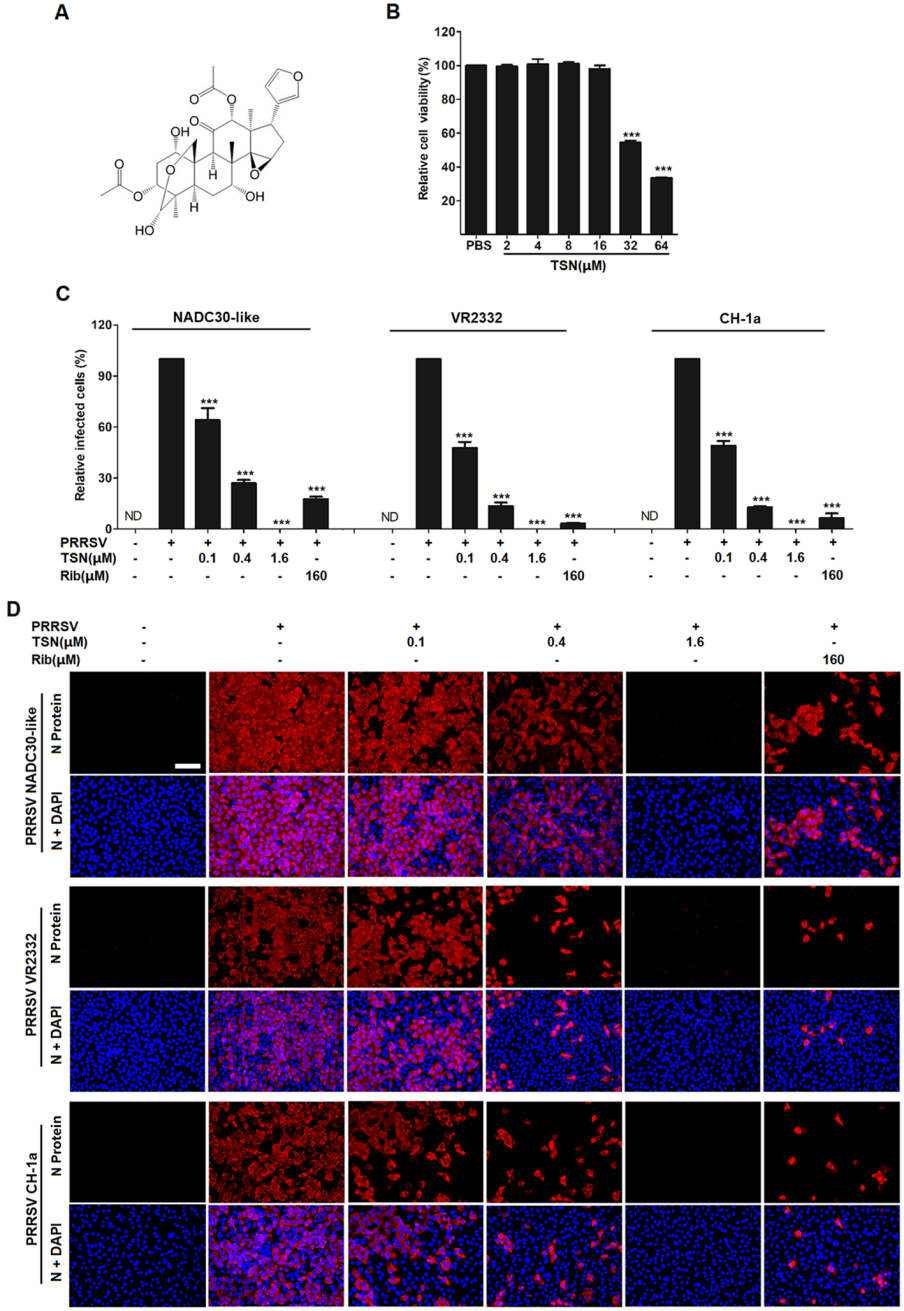
**Cytoxicity and anti-PRRSV activity of Toosendanin (TSN) in Marc-145 cells.** (**A**) Chemical structure of TSN. (**B**) TSN Cytoxicity in Marc-145 cells was examined using MTT assay and was presented as relative cell viability of the viable cells in the absence of the compound (set up as 100%). (**C**, **D**) TSN antiviral activity against PRRSV (NADC30-like, VR2332 and CH-1a) infection of Marc-145 cells was examined by immunofluorescence assays (IFA). Cells grown in 96-well plates were infected with PRRSV (0.05 MOI) for 2 h at 37 ℃ and then cultured in fresh media containing various concentrations of TSN. IFA for the N protein of PRRSV was performed at 48 hpi using Alexa Fluor 568-conjugated goat anti-mouse secondary antibody (red). Nuclei were counterstained using 4,6-diamidino-2-phenylindole (DAPI) (blue). Results shown in **C** are the mean values of percentages of PRRSV-infected cell ratio in TSN-treated groups compared to the DMSO-treated control (0 µM TSN, set as 100%) from three independent experiments; and **D** is one representative IFA data from **C**. Scale bar: 100 µm. Statistical significances are denoted by *p < 0.05, **p < 0.01, and ***p < 0.001.

The chemical structure of TSN is shown in Figure 1A. We first tested the cytotoxic effect of TSN on Marc-145 cells using MTT assay. As shown in Figure 1B, TSN did not affect Marc-145 cell viability when the concentration was increased to 16 μM. The 50% cytotoxic concentration (CC_50_) of TSN on Marc-145 cells was 42.32 μM. Next, we examined the antiviral effects of TSN against three different PRRSV strains (NADC30-like, VR-2333 and CH-1a). Marc-145 cells were infected by PRRSV for 48 h, fixed and then imaged by immunofluorescence microscopy. As shown in Figure 1C, D, NADC30-like, VR-2333 and CH-1a infections in Marc-145 cells were significantly inhibited by TSN in a dose-dependent manner. The half maximal effective concentrations (EC_50_) and the corresponding selectivity indexes (SI) were calculated based on the infected cell percentages to determine the antiviral activities of TSN on the three PRRSV strains. As shown in Table 2, the EC_50_ of TSN against the infections of NADC30-like, VR-2333 and CH-1a was 0.20, 0.18 and 0.16 μM, and the corresponding SI was 211, 235 and 264, respectively. These results indicate that TSN inhibited PRRSV infections in Marc-145 cells at concentrations far lower than its maximum non-cytotoxic concentration. Ribavirin, a well-known inhibitor of viral RNA polymerase, was reported to have anti-PRRSV activity [45] and thus used as the positive antiviral drug control in this study. Our results showed that 160 µM of ribavirin exhibited significant inhibition on the three PRRSV strains (Figure 1C, D). Strikingly, the inhibitory effects of TSN were overall stronger than those of ribavirin.

**Table 2:** **Inhibitory activity of Toosendanin (TSN) in Marc-145 cells infected by different PRRSV strains**

	PRRSV Strain		
	NADC30-like	VR-2332	CH-1a
EC_50_ (μM)^a^	0.20 ± 0.01	0.18 ± 0.01	0.16 ± 0.01
Selectivity index (SI)^b^	211	235	264

Data were presented as means ± SD of results from three independent experiments

^a^EC_50_, the concentration required to protect 50% cells from PRRSV infection by counting infected cells from IFA images, as described in the methods

^b^SI (selectivity index) is the ratio of CC_50_ to EC_50_. CC_50_ of TSN on Marc-145 cells was 42.32 µM

**Figure 2:**
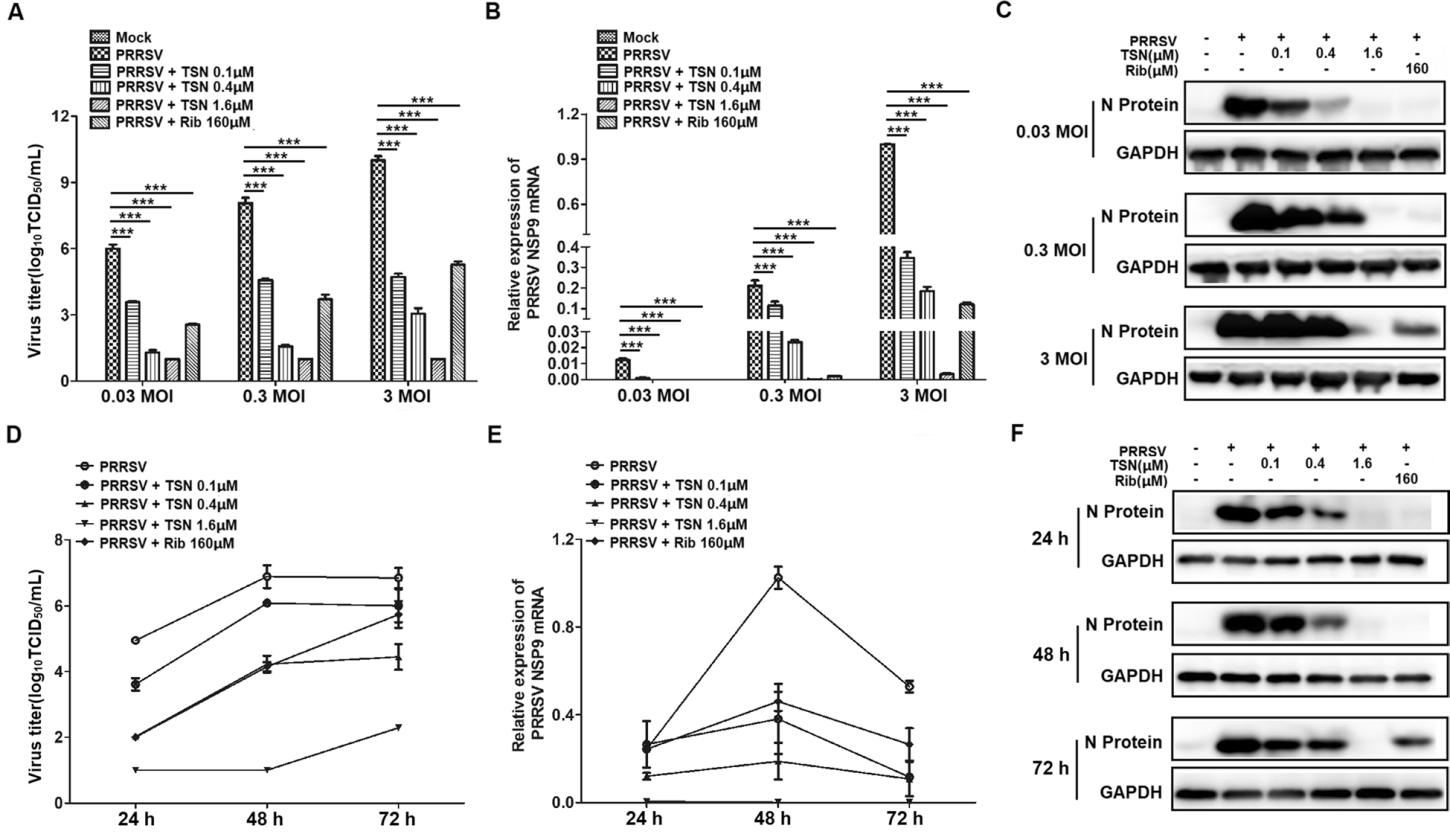
**Anti-PRRSV activity of TSN in Marc-145 cells.** (**A**–**C**) Cells grown in 6-well plates were infected with PRRSV NADC30-like (0.03, 0.3 and 3 MOI) for 2 h at 37 °C and then cultured in fresh media containing various concentrations of TSN. At 48 h post infection, the samples were subjected to viral titer (**A**), qRT-PCR (**B**), and Western blot (**C**) analyses. (**D**–**F**) Cells grown in 6-well plates were infected with PRRSV NADC30-like (0.05 MOI) for 2 h at 37 °C and then cultured in fresh media containing various concentrations of TSN. At indicated time-points post infection, the samples were subjected to viral titer (**D**), or qRT-PCR (**E**), or Western blotting analysis (**F**). Statistical significances are denoted by *p < 0.05, **p < 0.01, and ***p < 0.001.

We further examined the effects of TSN on PRRSV replication by infecting Marc-145 cells with different MOI (0.03, 0.3 and 3) of NADC30-like strain using virus titration, qRT-PCR, and Western blotting. As shown in Figure 2A, treatment with TSN resulted in a significant reduction of PRRSV titer in a dose-dependent manner at three different NADC30-like infection doses. Treatment with 1.6 µM of TSN led to 5, 7, 9 log reduction in progeny virus production compared to that in DMSO-treated control (Figure 2A). TSN at concentrations ranging from 0.1 to 1.6 µM significantly inhibited NADC30-like NSP9 mRNA and N protein levels in infected Marc-145 cells (Figure 2B, C). We next studied the PRRSV inhibition kinetics by three different concentrations of TSN from 24 to 72 hpi. In the infected control (PRRSV), the virus titer, viral N protein and viral NSP9 mRNA expressions increased markedly from 24 to 48 hpi, and viral NSP9 mRNA expressions decreased from 48 to 72 hpi (Figure 2D–F). The addition of TSN (0.1, 0.4 and 1.6 µM) significantly reduced virus titer, viral N protein and NSP9 mRNA levels at all time-points. The effects were more potent than 160 µM of Rib as shown in Figure 2D–F. Collectively, these data demonstrate that TSN significantly inhibits PRRSV NADC30-like replication in Marc-145 cells.

### TSN inhibits PRRSV infection in porcine alveolar macrophages

**Figure 3:**
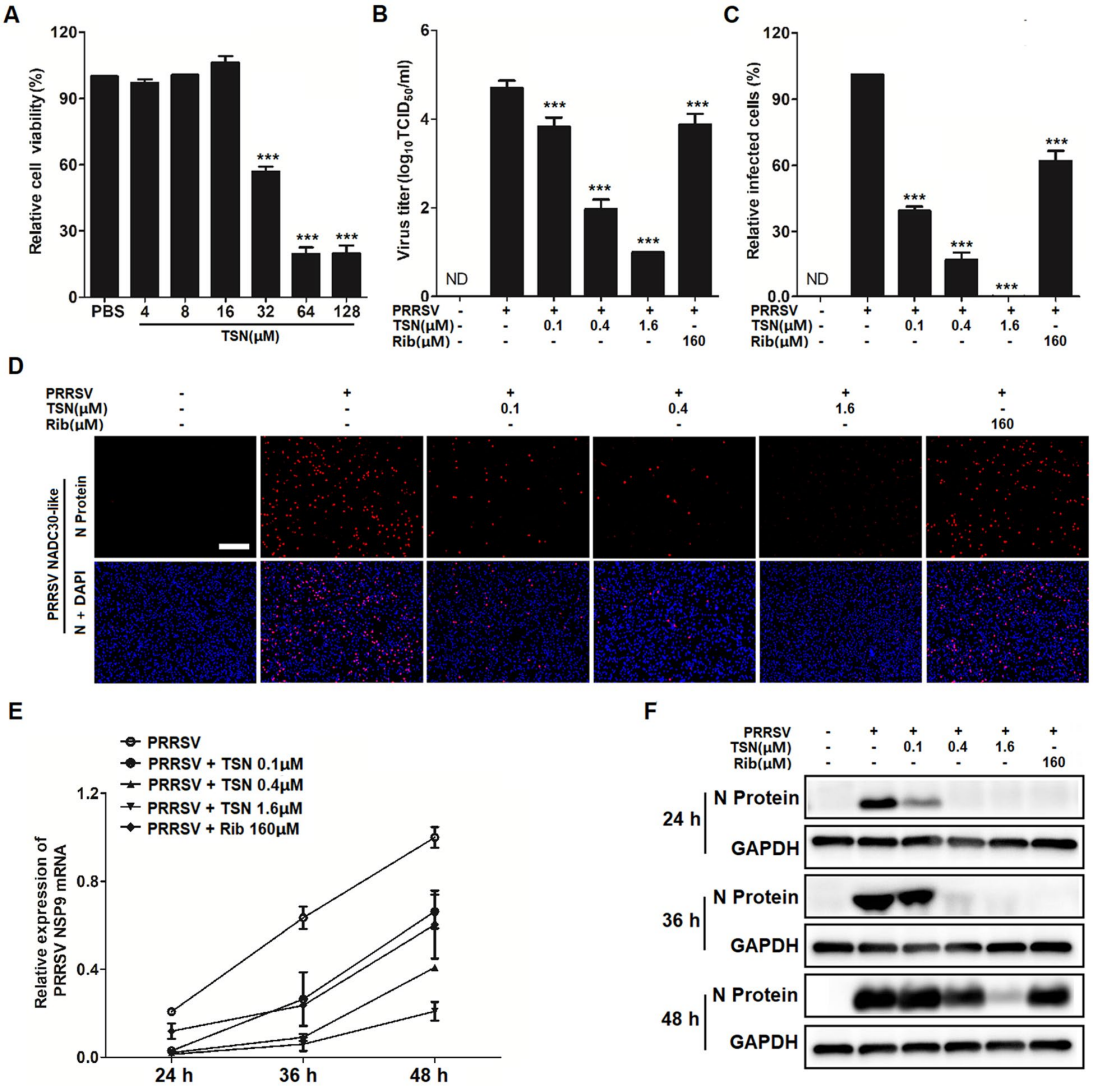
**TSN inhibits PRRSV replication in PAMs.** (**A**) TSN Cytoxicity in PAMs as examined using a MTT assay as described in the methods. (**B**–**D**) PAMs grown in 6-well plates (**B**) or 96-well plates (**C** and **D**) were infected with PRRSV NADC30-like (0.5 MOI) for 2 h at 37 °C and then treated with TSN at various concentrations. At 24 hpi, the samples were subjected to viral titer (**B**), and IFA (**C** and **D**) analyses. Results shown in (**C**) are the mean percentage values of PRRSV-infected cell ratio in TSN-treated groups compared to the DMSO-treated control (0 μM TSN, set as 100%) from three independent experiments, and (**D**) is one representative IFA data from (**C**). (**E** and **F**) Cells grown in 6-well plates were infected with PRRSV NADC30-like (0.5 MOI) for 2 h at 37 °C and then cultured in fresh media containing various concentrations of TSN. At the indicated time-points post infection, the samples were subjected to qRT-PCR (**E**), or Western blotting (**F**) analysis. Statistical significances are denoted by *p < 0.05, **p < 0.01, and ***p < 0.001.

Since TSN exerted a potent antiviral activity against PRRSV infection in Marc-145 cells, we therefore investigated whether TSN could also inhibit PRRSV ex vivo replication in PAMs, the major target cell type of PRRSV infection in pigs. We first assessed TSN cytotoxicity on PAMs using MTT assay. As shown in Figure 3A, TSN exhibited a cytotoxicity profile on PAMs like that on Marc-145 cells, with 16 µM of TSN found non-cytotoxic to PAMs and the CC_50_ determined as 31.49 µM. Next, we evaluated the antiviral effects of TSN against PRRSV NADC30-like replication in PAMs using virus titration and IFA. As shown in Figure 3B–D, TSN significantly reduced progeny virus production and N protein level; treatment with 1.6 µM of TSN resulted in a 3.8 log reduction in progeny virus production when compared to that in the DMSO control (Figure 3B). Inhibition of TSN on NADC30-like replication in PAMs from 24 to 48 hpi was also observed by qRT-PCR and Western blotting. As shown in Figure 3E, F, the addition of 1.6 µM of TSN significantly reduced viral NSP9 mRNA and N protein levels at all time-points. Again, the effects were more potent than 160 µM of Rib. Taken together, TSN could effectively inhibit PRRSV NADC30-like infection in ex vivo PAMs.

### TSN does not interact directly with PRRSV

**Figure 4:**
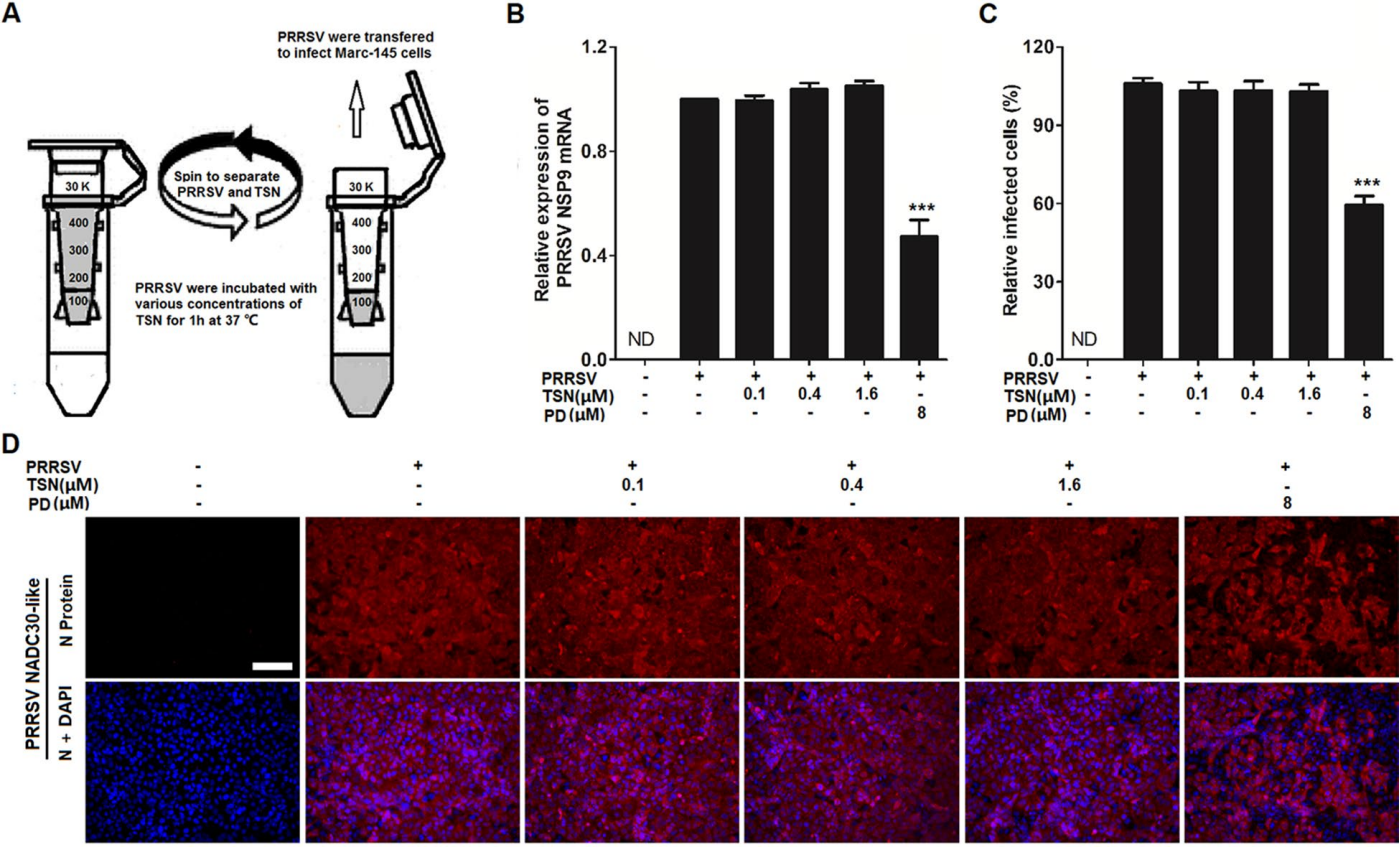
**TSN does not directly interact with PRRSV.** 100 µL of PRRSV NADC30-like (5 MOI) was mixed with various concentrations of TSN in essential media (0.9 mL total volume) for 1 h at 37 ℃. Then PRRSV and TSN were separated by ultrafiltration, as shown in (**A**). Recovered PRRSV were resuspended to infect Marc-145 cells. 48 h later, the samples were analyzed by qRT-PCR or IFA. (**B**) Relative viral NSP9 mRNA expression of TSN treated groups to DMSO-treated control (set as 1) was analyzed using qRT-PCR. Results shown in (**C**) are the mean values of percentage of PRRSV-infected cell ratio in TSN-treated groups compared to the DMSO-treated control (0 μM TSN, set as 100%) from three independent experiments, and (**D**) is one representative IFA data set from (**C**). Scale bar: 100 µm. Statistical significances are denoted by *p < 0.05, **p < 0.01, and ***p < 0.001.

As demonstrated above, TSN exerted a remarkable antiviral activity against PRRSV infection in Marc-145 cells and PAMs. We thus questioned whether TSN could inactivate PRRSV by directly interacting with the virus, thereby inhibiting virus infection. To address this question, TSN (at final concentrations of 0.1, 0.4 and 1.6 μM) was mixed with PRRSV NADC30-like, incubated for 1 h at 37 ℃. Then PRRSV and TSN were separated by ultrafiltration, as shown in Figure 4A. Recovered PRRSV were resuspended to infect Marc-145 cells. At 48 hpi, the cells were subjected to viral mRNA expression and PRRSV-infected cell ratio analysis using qRT-PCR and IFA, respectively. As shown in Figure 4B–D, co-incubation of TSN (0.1, 0.4, and 1.6 µM) with the virus did not weaken PRRSV ability to infect Marc-145 cells, demonstrating that TSN does not directly interact with PRRSV particles. Platycodin D (PD), a triterpenoid saponin, which was reported to inhibit PRRSV replication by directly interacting with the virions, was used as positive control in this study [11].

### TSN pretreatment significantly reduces PRRSV replication

**Figure 5:**
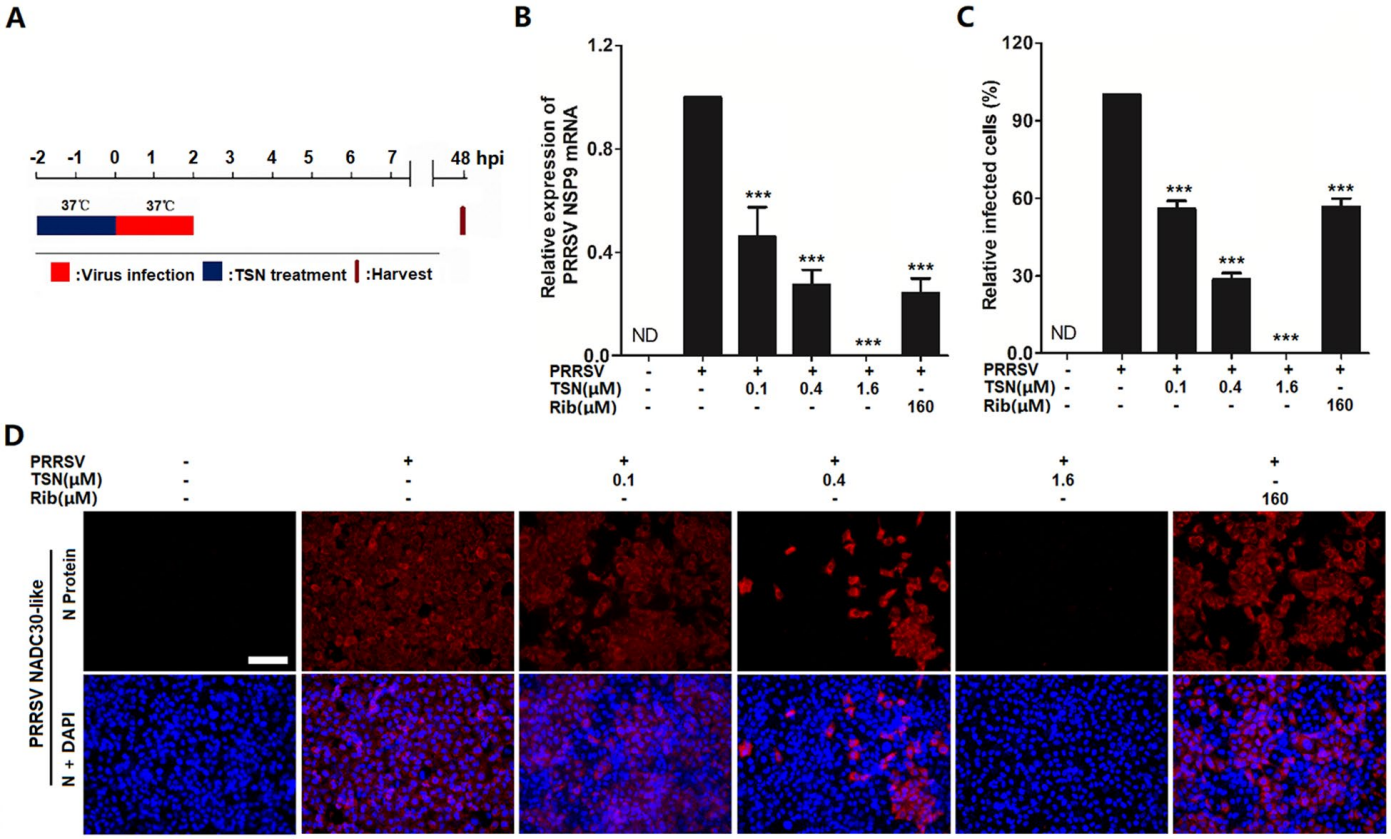
**TSN pretreatment of Marc-145 cells suppresses PRRSV replication**. Marc-145 cells were treated with 0.1, 0.4 or 1.6 μM TSN for 2 h prior to being infected with PRRSV NADC30-like. 48 h later, the samples were subjected to qRT-PCR (**B**), or IFA (**C** and **D**). Results shown in **C** are the mean values of percentages of PRRSV-infected cells in TSN-treated groups compared to that in the DMSO-treated control (0 µM TSN, set as 100%) from three independent experiments, and (**D**) is one representative IFA data set from (**C**). Scale bar: 100 µm. Statistical significances are denoted by * *p* < 0.05, **p < 0.01, and ***p < 0.001.

Having excluded TSN-virus direct interaction, we wanted to identify whether TSN could potentially alter the cell susceptibility to PRRSV. Marc-145 cells were treated with 0.1, 0.4 or 1.6 μM of TSN before infection (Figure 5A). As shown in Figure 5B–D, the level of viral mRNA and N protein were significantly reduced by TSN pretreatment; pretreatment with 1.6 µM of TSN resulted in a 100% protection from PRRSV infection. These results indicate that TSN interacts with cellular components to reduce PRRSV replication.

### TSN treatment upregulates IFI16 expression in Marc-145 cells

**Figure 6:**
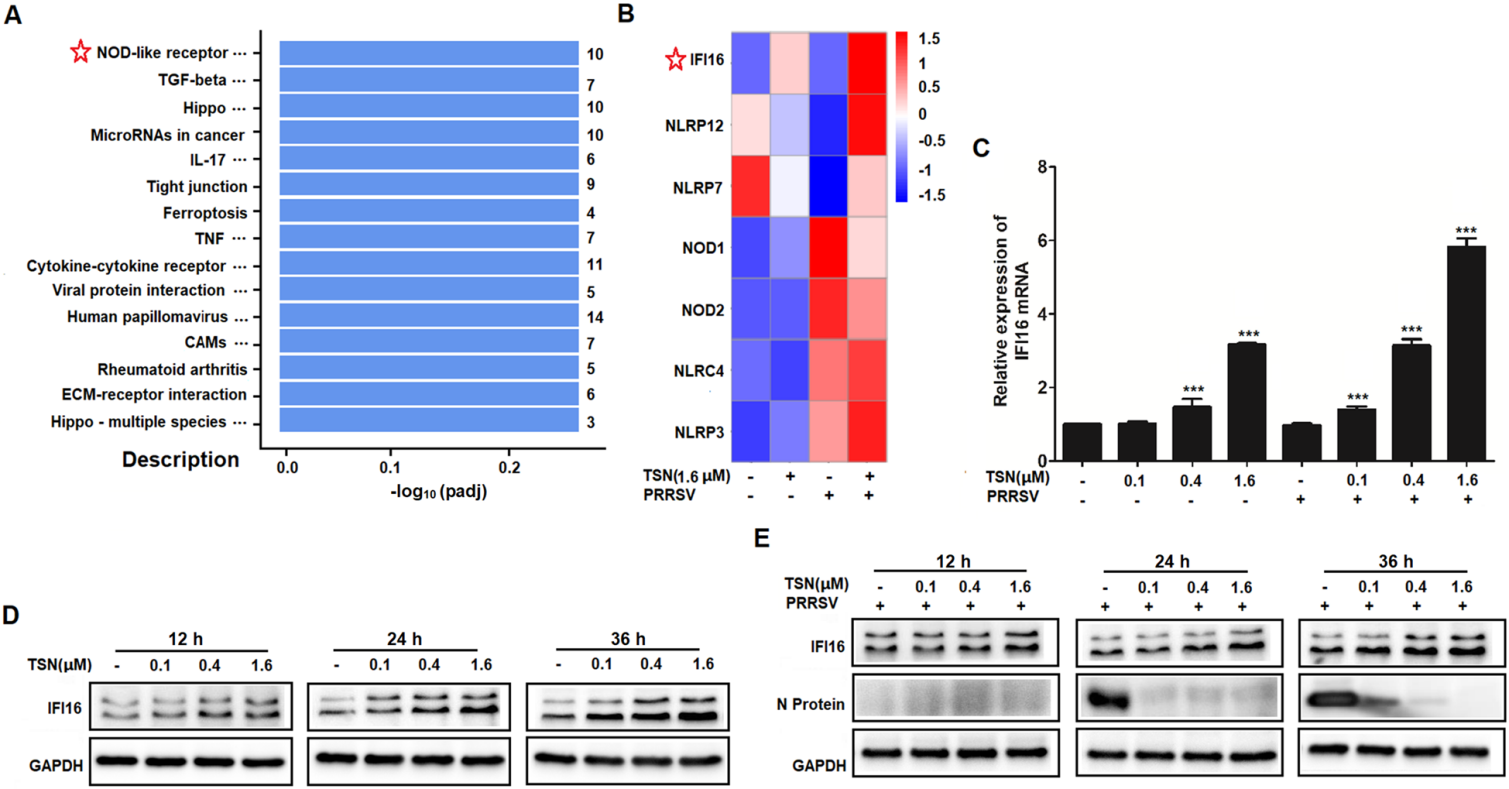
**TSN treatment upregulates cellular IFI16 expression and suppresses PRRSV replication in Marc-145 cells.** (**A** and **B**) Marc-145 cells grown in 6-well plates were infected or not infected with PRRSV NADC30-like (0.05 MOI) for 2 h at 37 °C and then cultured in fresh medium containing 1.6 μM TSN. At 4 h post infection, the samples were subjected to high-throughput RNA sequencing. (**A**) The clusterProfiler R package was used to test the statistical enrichment of differentially expressed genes in KEGG pathways. (**B**) Heat map of expressed NOD-like pathway genes modulated by 1.6 μM TSN or DMSO treatment in Marc-145 cells. Red and blue correspond to relative up- and down-regulation, respectively. (**C**) Cells grown in 12-well plates were infected or not infected with PRRSV NADC30-like (0.05 MOI) for 2 h at 37 °C and then cultured in fresh media containing various concentrations of TSN. At 24 hpi, the samples were subjected to qRT-PCR analysis. (**D** and **E**) Cells grown in 6-well plates were not infected (**D**) or infected (**E**) with PRRSV NADC30-like (0.05 MOI) for 2 h at 37 °C and then cultured in fresh media containing various concentrations of TSN. The samples were collected and total protein were extracted from cell lysates at 12, 24 and 36 hpi, respectively. Western blotting was used to analyze IFI16 and GAPDH expression. Statistical significances are denoted by *p < 0.05, **p < 0.01, and ***p < 0.001.

To investigate the mechanism underlying TSN anti-PRRSV effect, we performed high-throughput RNA sequencing (RNA-seq) to explore gene expression in Marc-145 cells that had been treated with or without TSN for 2 h. KEGG analysis showed that differentially expressed genes (DEGs) were significantly enriched in the NOD-like receptor signaling pathway, as marked by asterisks (Figure 6A). We therefore investigated a large set of genes associated with the NOD-like receptor signaling pathway, including NOD1, NOD2, NLRC4, NLRC12, NLRP7, NLRP3 and IFI16. Notably, our results unveiled that TSN upregulated the expression of IFI16 in both uninfected and infected Marc-145 cells (Figure 6B), indicating that the protective effects of TNS against PRRSV infection might be associated with enhanced IFI16 expression. To confirm the results of the RNA-seq analysis, the effects of TSN on IFI16 mRNA expression were measured by qRT-PCR at 24 hpi. As shown in Figure 6C, TSN significantly upregulated IFI16 mRNA expression in both uninfected and infected Marc-145 cells. To further explore the role of TSN-induced IFI16 in PRRSV-infected cells, uninfected (Figure 6D) and infected (Figure 6E) Marc-145 cells were treated with TSN at various concentrations for 12 h, 24 h or 36 h, followed by Western blot analysis for IFI16 protein expression. The results showed that TSN gradually upregulated IFI16 protein expression in both uninfected and infected cells as the treatment time increased. Previous studies highlight IFI16 as a critical antiviral factor during virus infection [18, 46], we speculated that the inhibition of TSN on PRRSV replication was related to the upregulation of IFI16 protein expression.

### TSN inhibits PRRSV replication via up-regulating IFI16 expression

**Figure 7:**
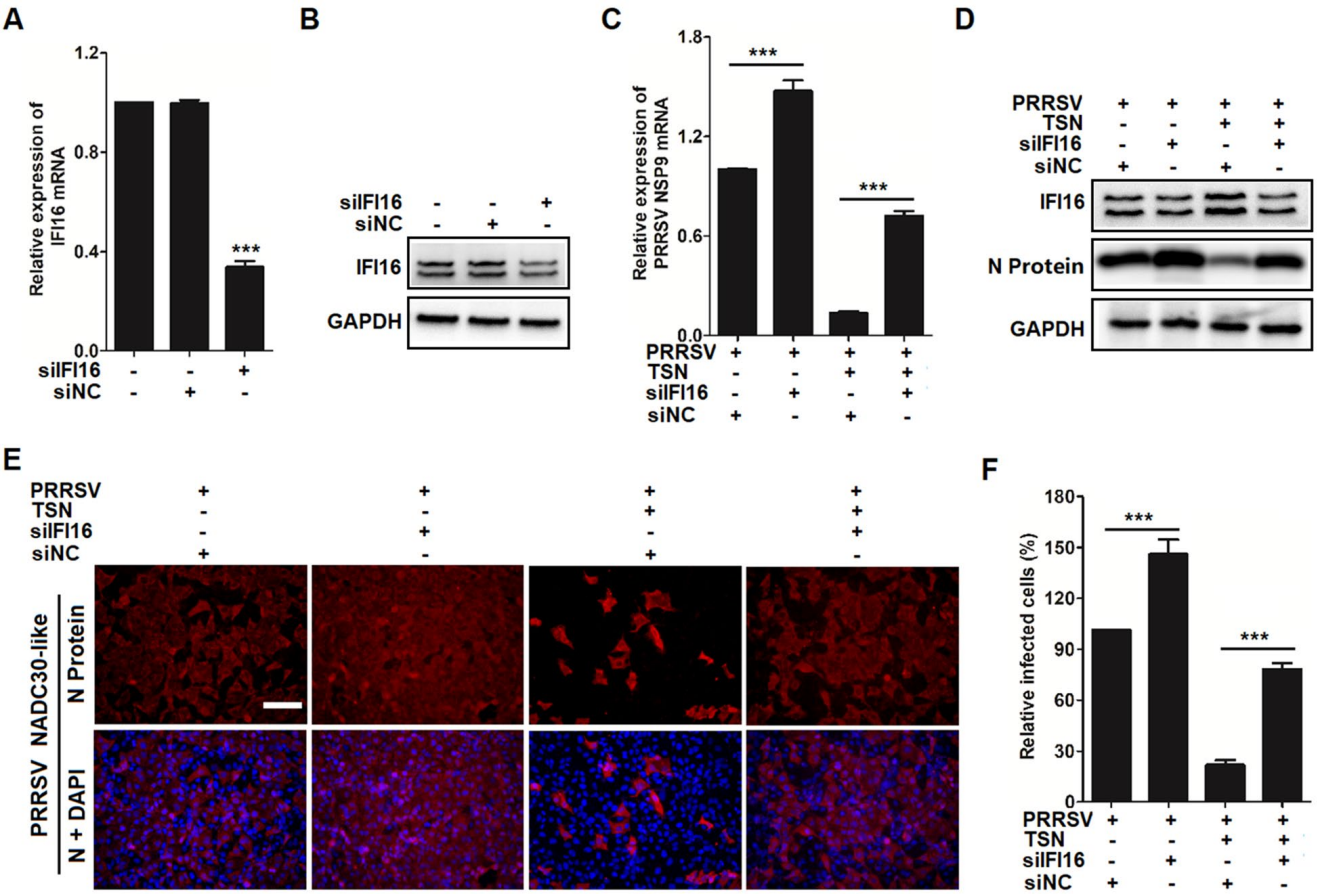
**TSN upregulates IFI16 to inhibit PRRSV replication.** (**A** and **B**) Marc-145 cells were transfected with siRNA targeting IFI16 and siNC. Total RNA and protein of the samples were extracted and subjected to IFI16 mRNA (**A**) and protein (**B**) analysis using qRT-PCR and Western blotting, respectively. (**C**–**F**) Marc-145 cells were transfected with siRNA targeting IFI16 or siNC. At 24 h post-transfection (hpt), the transfected cells were infected with PRRSV (0.05 MOI) for 2 h at 37 ℃ and then cultured in fresh medium containing 0.4 μM TSN. At 24 hpi, the samples were subjected to qRT-PCR (**C**), Western blotting (**D**), or IFA (**E** and **F**) analysis. Scale bar: 100 μm. Statistical significances are denoted by *p < 0.05, **p < 0.01, and ***p < 0.001.

Since TSN upregulated IFI16 expression in both PRRSV-infected and -uninfected Marc-145 cells, we questioned whether this upregulation may contribute to TSN anti-PRRSV activity. We therefore designed specific siRNAs to inhibit IFI16 expression (Figure 7A, B). Silencing IFI16 resulted in enhanced PRRSV replication as shown by the increased levels of viral NSP9 mRNA in Figure 7C. Similarly, viral N protein was also markedly increased following IFI16 silencing (Figure 7D–F). Importantly, we found that IFI16 silencing impacted TSN anti-PRRSV activity reflected by increased PRRSV replication (Figure 7C), a 56% increase in viral N protein expression (Figure 7E, F). These results indicate that TSN inhibits PRRSV replication via up-regulating IFI16 expression.

### TSN enhances caspase-1-mediated maturation of IL-1β to inhibit PRRSV replication via an IFI16-dependent pathway in Marc-145 cells

**Figure 8:**
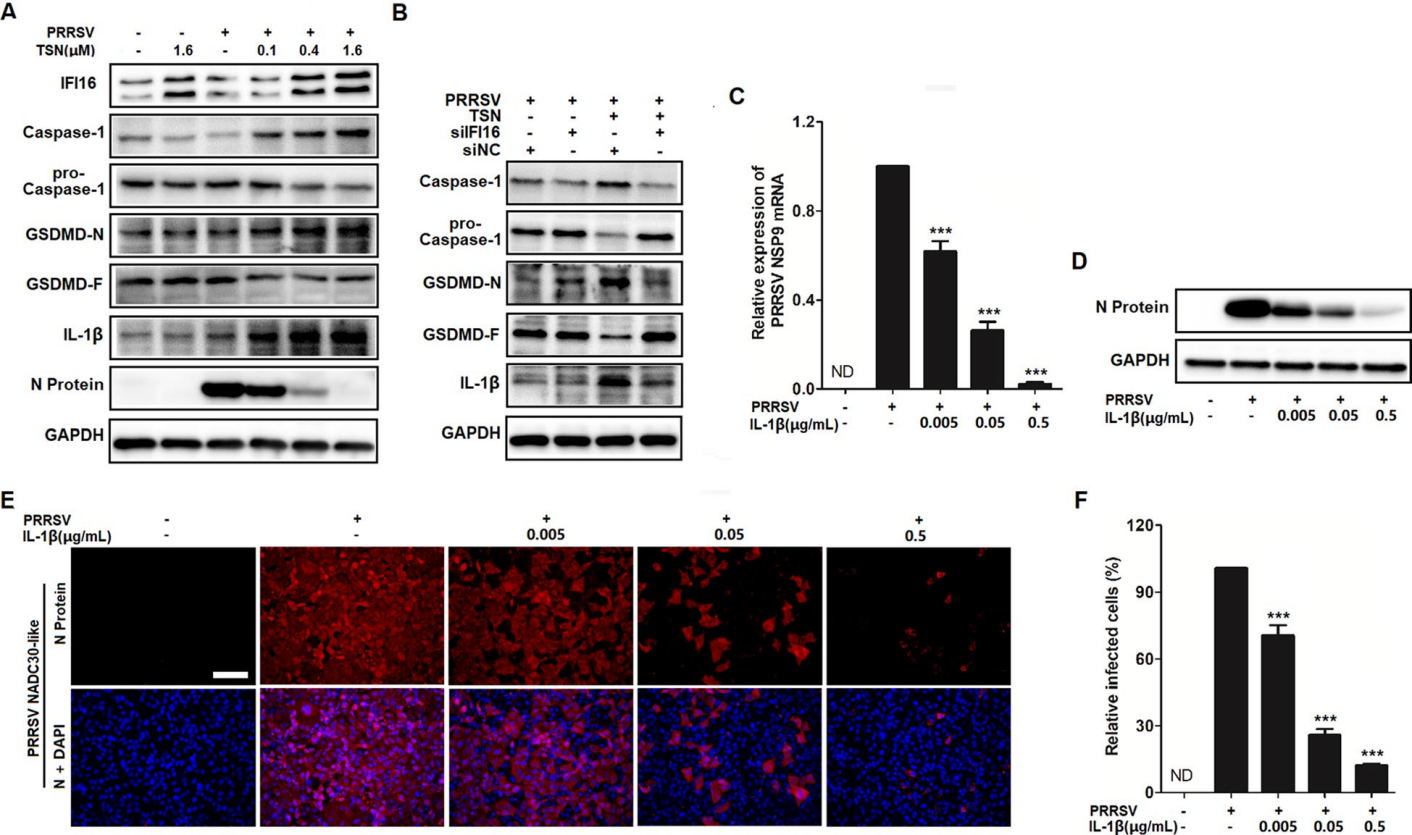
**TSN enhances the production of IL-1β to inhibit the replication of PRRSV.** (**A**) Marc-145 cells grown in 6-well plates were infected with PRRSV NADC30-like (0.05 MOI) for 2 h at 37 ℃ and then cultured in fresh media containing various concentrations of TSN. (**B**) Marc-145 cells were transfected with siRNA targeting IFI16. At 24 hpt, the transfected cells were infected with PRRSV at 0.05 MOI and then cultured in fresh media containing TSN (0.4 μM). After 24 h, the cells were harvested for Western blot analyses to detect the level of IFI16, caspase-1, pro-caspase-1, GSDMD-N, GSDMD-F, IL-1β, viral N protein and GAPDH. (**C**–**F**) Marc-145 cells grown in 24-well plates were infected with PRRSV NADC30-like (0.05 MOI) for 2 h at 37 ℃ and then cultured in fresh media containing various concentrations of IL-1β. At 24 hpi, the samples were subjected to qRT-PCR (**C**), Western blotting (**D**), or IFA (**F**). Scale bar: 100 μm. Statistical significances are denoted by *p < 0.05, **p < 0.01, and ***p < 0.001.

DNA recognition of pathogenic microorganisms by IFI16 leads to the activation of the caspase-1-dependent inflammasome and the maturation of IL-1β [27]. To identify the role of caspase-1-dependent inflammasome in the inhibition of PRRSV infection by TSN, Marc-145 cells were infected with PRRSV and the PRRSV N protein and a set of proteins in the casepase-1 signaling pathway, including caspase-1, pro-caspase-1, GSDMD-N, GSDMD-F and IL-1β were analyzed by Western blotting. As shown in Figure 8A, there was no significant change in the expressions of those casepase-1 regulated proteins following PRRSV infection, while TSN treatment significantly upregulated the expression of caspase-1 and its downstream target proteins (GSDMD-N, IL-1β). Meanwhile, a decrease in virus replication, reflected by attenuated N-protein expression, was also observed in TNS-treated infected cells. To further identify the effect of IFI16-driven activation on the caspase-1 signaling pathway, Marc-145 cells were transfected with IFI16 siRNA or negative siRNA (siNC) for 24 h followed by PRRSV infection and then TSN treatment (0.4 μM). As shown in Figure 8B, IFI16 silencing decreased the overexpression of caspase-1, GSDMD-1 and IL-1β induced by TSN treatment in PRRSV-infected Marc-145 cells.

Since IL-1β plays an important role in antiviral host defense [46], we therefore investigated the antiviral effect of IL-1β against PRRSV infection. For this purpose, Marc-145 cells were infected with PRRSV NADC30-like (0.05 MOI) for 2 h at 37 ℃ and then cultured in fresh media containing various concentrations of IL-1β for 24 h followed by qRT-PCR, Western blotting and IFA. As expected, exogenous IL-1β significantly reduced viral NSP9 mRNA and N protein levels in Marc-145 cells (Figure 8C, D), treatment with 0.5 µg/mL of IL-1β resulted in an 88% reduction of viral N protein when compared to that in the DMSO control (Figure 8E, F). These data together demonstrate that TSN enhances caspase-1-mediated production of IL-1β to inhibit PRRSV replication through an IFI16-dependent pathway.

### TSN inhibits PRRSV replication in PAMs via promoting the IFI16/caspase-1/IL-1β pathway

**Figure 9:**
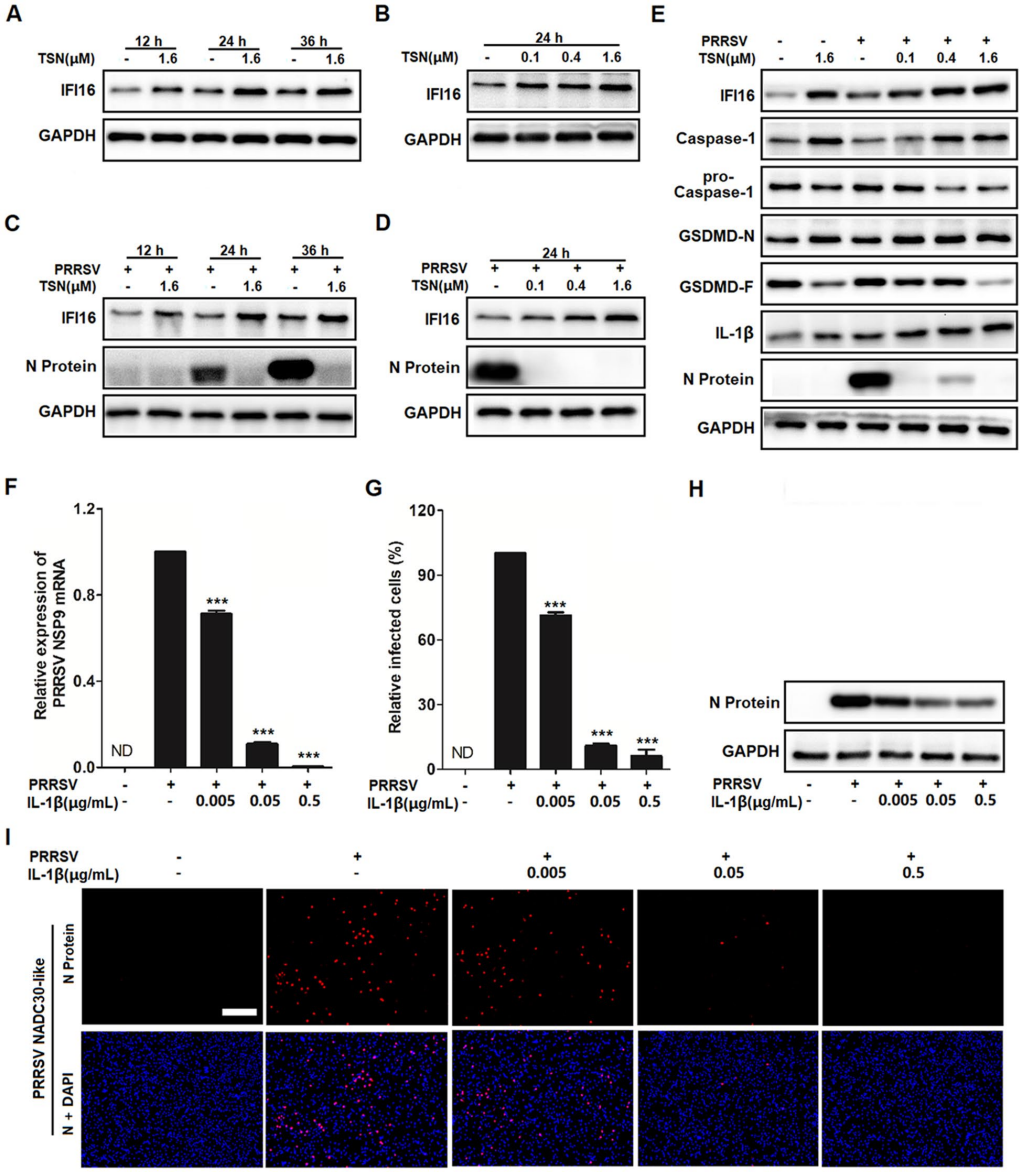
**TSN promotes the IFI16/Caspase-1/IL-1β pathway to inhibit PRRSV replication in PAMs.** (**A**–**D**) PAMs grown in 12-well plates were not infected (**A** and **B**) or infected (**C** and **D**) with PRRSV NADC30-like (0.5 MOI) for 2 h at 37 ℃ and then cultured in fresh media containing various concentrations of TSN. Total protein was extracted from cell lysates at 12, 24 and 36 h, respectively. The expression levels of IFI16, GAPDH and viral N protein were analyzed by Western Blotting. (**E**). PAMs grown in 6-well plates were not infected or infected with PRRSV NADC30-like (0.5 MOI) for 2 h at 37 ℃ and then cultured in fresh medium containing various concentrations of TSN. At 24 hpi, the samples were subjected to Western blot analyses to measure the levels of IFI16, caspase-1, pro-caspase-1, GSDMD-N, GSDMD-F, IL-1β, viral N protein and GAPDH. (**F**-**I**) Cells grown in 24-well plates were infected with PRRSV NADC30-like (0.5 MOI) for 2 h at 37 ℃ and then cultured in fresh medium containing various concentrations of IL-1β. At 24 hpi, the samples were subjected to qRT-PCR (**F**), Western Blotting (**H**) and IFA (**G** and **I**). Scale bar: 100 μm. Statistical significances are denoted by *p < 0.05, **p < 0.01, and ***p < 0.001.

Given that PAMs are natural target cells in pigs, we investigated whether TSN enhances caspase-1-mediated IL-1β production by up-regulating IFI16, thereby inhibiting PRRSV replication in PAMs. PRRSV-infected and uninfected PAMs were treated with 1.6 μM of TSN. At the indicated time points, the expression levels of IFI16 and N protein were analyzed by Western blotting. As shown in Figure 9A–D, TSN continuously induced the expression of IFI16 and inhibited PRRSV replication (reflected by reduced N protein expression) in PAMs. TSN also induced overexpression of caspase-1, GSDMD-N, and IL-1β in uninfected and in PRRSV-infected PAMs in a dose-dependent manner (Figure 9E), as in Marc-145 cells. Finally, we explored whether IL-1β could also inhibit PRRSV replication in PAMs. As shown in Figure 9F-I, IL-1β treatment significantly inhibited PRRSV replication in a dose-dependent manner in PAMs, as indicated by the reduced levels of viral NSP9 mRNA and N protein. At 24 hpi, treatment with 0.5 µg/mL of IL-1β resulted in an 92% reduction of viral N protein when compared to that in the DMSO control (Figure 9G, I). These data demonstrate that TSN promotes caspase-1-mediated maturation of IL-1β to inhibit PRRSV replication in PAMs also via the IFI16-dependent pathway.

## Discussion

Toosendanin (TSN), a natural triterpenoid compound, was initially extracted from the bark of *Melia toosendan* Seib. et Zucc. (a traditional Chinese medicine) in the 1950’s and was then used as an ascarifuge in China [31]. Previous research indicated that TSN possesses broad biological activities, such as blocking acetylcholine release, and anti-botulismic, and anti-tumor effects [47, 48]. Importantly, TSN was reported to inhibit infections of viruses such as HCV [38], IAV [37], SFTSV and SARS-CoV-2 [39]. However, these studies mainly focused on TSN antiviral activities rather than its mechanisms of action. The present study is the first to show that TSN potently inhibits PRRSV infection at sub-micromolar concentrations in Marc-145 cells and in ex vivo PAMs. Mechanistically, we demonstrate that TSN induces IFI16 expression, which leads to increased expression of caspase-1 and IL-1β, the later contributing to the inhibition of PRRSV replication in the infected cells.

As TSN exhibited a potent inhibitory activity against PRRSV at very low concentrations in Marc-145 cells and PAMs (Figures 1, 2, 3), we questioned whether TSN could inactivate PRRSV by directly interacting with virus particles, thereby inhibiting the infection. To address this question, we designed a series of experiments (Figures 4 and 5). Surprisingly, pretreatment of TSN with cells, rather than with viruses, significantly reduced PRRSV replication (Figure 5), suggesting that TSN may act on the cells to inhibit PRRSV infection but not directly inactivate PRRSV. This prompts us to perform a transcriptome analysis on PRRSV-infected and uninfected Marc-145 cells that were treated with or without TSN. Of the five hundred thirty-three differentially regulated genes, IFI16 was one of the most significantly upregulated genes in TSN-treated PRRSV-infected Marc-145 cells. The upregulation of IFI16 was also observed in TSN-treated uninfected Marc-145 cells (Figures 6A, B). IFI16 is a member of the pyrin and HIN domain (PYHIN) containing protein family, which includes key mediators of the innate immune response that sense microbial DNAs to induce IFNs and/or inflammasome activation [18, 49]. Previous studies have shown that IFI16 inhibits human cytomegalovirus (HCMV) transcription [19] and restricts herpes simplex virus 1 (HSV-1) replication [20]. In recent years, the role of IFI16 in RNA virus infections has been identified, whereby IFI16 transcriptionally upregulates the gene expression of IFN-I in Sendai virus (SeV) and mouse hepatitis coronavirus infection [22, 50, 51]. More recently, Wichit et al. found that enhanced expression of IFI16 completely restricted Chikungunya virus (CHIKV) infection while endogenous silencing of the gene markedly increased virus replication [52]. These findings suggest that IFI16 is a key positive regulator in antiviral innate immune responses. However, inducers for IFI16 were barely reported. The present study shows that TSN inhibit PRRSV replication via enhancing IFI16 expression in Marc-145 cells (Figure 6C–E) and PAMs (Figure 9A–D). More importantly, these effects were reversed by silencing the IFI16 gene (Figures 7 and 8), corroborating that the anti-PRRSV activity of TSN is IFI16-dependent. Thus, our findings demonstrate that TSN is a potent inducer of the IFI16 and might serve as a therapeutic agent against PRRSV infection.

**Figure 10:**
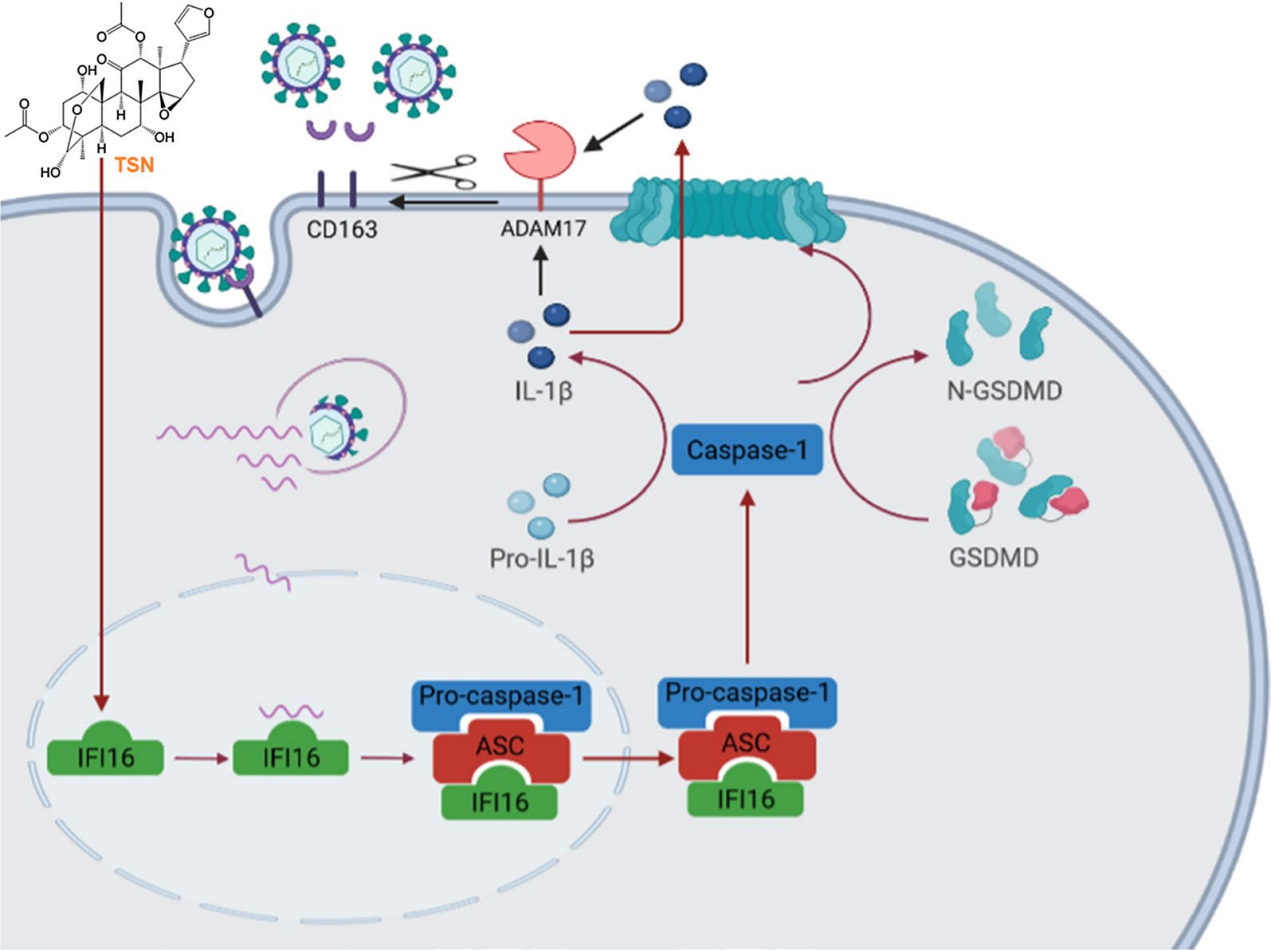
**Scheme summarizing the inhibitory effect of TSN on PRRSV replication via activation of caspase-1 and induced maturation of IL-1β in an IFI16-dependent pathway**. TSN significantly upregulates IFI16 expression in Marc-145 cells and PAMs. During primary infection of Marc-145 cells or PAMs, PRRSV nucleocapsid travels to the nuclear pore, and the linear RNA enters the nucleus, which is sensed by IFI16 during latency. This leads to the recruitment of ASC and pro-caspase-1 to form an inflammasome complex; the complex subsequently translocates to the cytoplasm to activate caspase-1, which then catalyzes the maturation of IL-1β. Eventually, the activated IL-1β are sorted and released from cells to inhibit PRRSV.

IFI16 inflammasome is a large signaling platform activated by IFI16 in response to viral infection, which also contains the adapter protein ASC (apoptosis-associated speck-like protein containing a caspase recruitment domain) and effector enzyme pro-caspase-1 [27, 53]. Inflammasome activation was examined by degradation of pro-caspase-1 to caspase-1 [54]. IL-1β is generated via proteolytic cleavage of pro-IL-1β by caspase-1 during inflammasome activation [55, 56]. Here, we demonstrated that TSN displayed anti-PRRSV activity by activating IFI16 inflammasome. TSN treatment enhanced expressions of caspase-1, GSDMD-N and IL-1β in a dose-dependent manner in PRRSV-infected Marc-145 cells and PAMs (Figures 8A and 9E), and these effects were reversed by silencing the IFI16 gene, indicating that TSN enhances caspase-1-mediated maturation of IL-1β to inhibit PRRSV replication in an IFI16-dependent pathway. IL-1β is an important component of the host defense against viral infection, due to its key role in innate immunity [57]. There is ample evidence showing that the activation of inflammasomes in the airway epithelium and concomitant mucosal production of IL-1β and IL-18 are crucial for optimal antibody and T cell responses against IAV [29, 58, 59]. In addition, Li et al. found that downregulation of pMGF505-7R expression induces higher levels of IL-1β production which lowers pathogenicity in pigs with ASFV infection, and enhance viral clearance and infected-host survival [30, 60]. Song et al. found that 25-Hydroxycholesterol significantly decreased the replication of PRRSV, and increased the production of IL-1β and IL-8 in porcine primary alveolar macrophages and the lung tissue [61]. In this work, we extend IL-1β inhibiting role on PRRSV replication (Figures 8C–F and 9F-I). It can be concluded that TSN inhibits PRRSV infection partly by up-regulating the expression of IL-1β. Recently, Lauren et al. showed that IL-1β induced antiviral interferon responses against West Nile virus infection in dendritic cells through a process of IL-1β-to-IRF3 signaling crosstalk [62]. In addition, Lanaya et al. showed that IL-1β stimulation of Kupffer cells enhanced ADAM17 expression at both transcriptional and proteic levels [26]. ADAM17 is one of the important membrane-associated metalloproteases that mediates various cellular events as well as inflammation, cancer, and other pathologies [63]. Guo et al. confirmed that ADAM17 is able to suppress PRRSV entry by down-regulating the expression of membrane CD163, which is an essential viral receptor mediating PRRSV entry and uncoating [64]. A schematic summary for the inhibitory cascade of TSN on PRRSV replication is proposed in Figure 10. However, the exact mechanism by which elevated IL-1β suppresses PRRSV replication needs to be further investigated.

In our study, TSN exhibited potent inhibition against PRRSV replication at concentrations ranging from 0.1 to 1.6 μM in vitro, raising the question of whether such concentrations could be attainable in pigs when TSN is administrated at safe doses. Wang et al. reported that the average peak plasma drug concentrations of TSN in rat (single 2 mg/kg of body weight intravenous injection) range from 1.5 to 7.76 μM [65], which are much higher than the effective anti-PRRSV concentrations in our in vitro experiments. It is therefore reasonable to speculate that therapeutical TSN concentrations are reachable in PRRSV-infected pigs.

In summary, our study shows that TSN potently suppresses PRRSV replication at sub-micromolar concentrations. Furthermore, we elucidate the mechanisms underlying antiviral effect of TSN against PRRSV infections as activating caspase-1 to induce IL-1β maturation via IFI16-inflammasome. TSN therefore has the potential to become a novel antiviral agent.

## References

[1] An T, Li J, Su C, Yoo D (2020). Molecular and cellular mechanisms for PRRSV pathogenesis and host response to infection. Virus Res.

[2] Lunney JK, Fang Y, Ladinig A, Chen N, Li Y, Rowland B, Renukaradhya GJ (2016). Porcine reproductive and respiratory syndrome virus (PRRSV): pathogenesis and interaction with the immune system. Annu Rev Anim Biosci.

[3] Neumann EJ, Kliebenstein JB, Johnson CD, Mabry JW, Bush EJ, Seitzinger AH, Green AL, Zimmerman JJ (2005). Assessment of the economic impact of porcine reproductive and respiratory syndrome on swine production in the United States. J Am Vet Med Assoc.

[4] Snijder EJ, Kikkert M, Fang Y (2013). Arterivirus molecular biology and pathogenesis. J Gen Virol.

[5] Tong T, Hu H, Zhou J, Deng S, Zhang X, Tang W, Fang L, Xiao S, Liang J (2020). Glycyrrhizic-acid-based carbon dots with high antiviral activity by multisite inhibition mechanisms. Small.

[6] Dokland T (2010). The structural biology of PRRSV. Virus Res.

[7] Song J, Shen D, Cui J, Zhao B (2010). Accelerated evolution of PRRSV during recent outbreaks in China. Virus Genes.

[8] Long F, Zhang M, Yang X, Liang X, Su L, An T, Zhang G, Zeng Z, Liu Y, Chen W, Chen J (2021). The antimalaria drug artesunate inhibits porcine reproductive and respiratory syndrome virus replication via activating AMPK and Nrf2/HO-1 signaling pathways. J Virol.

[9] Duan E, Wang D, Fang L, Ma J, Luo J, Chen H, Li K, Xiao S (2015). Suppression of porcine reproductive and respiratory syndrome virus proliferation by glycyrrhizin. Antiviral Res.

[10] Liu X, Song Z, Bai J, Nauwynck H, Zhao Y, Jiang P (2019). Xanthohumol inhibits PRRSV proliferation and alleviates oxidative stress induced by PRRSV via the Nrf2-HMOX1 axis. Vet Res.

[11] Zhang M, Du T, Long F, Yang X, Sun Y, Duan M, Zhang G, Liu Y, Em Z, Chen W, Chen J (2018). Platycodin D suppresses type 2 porcine reproductive and respiratory syndrome virus in primary and established cell lines. Viruses.

[12] Ge M, Xiao Y, Chen H, Luo F, Du G, Zeng F (2018). Multiple antiviral approaches of (-)-epigallocatechin-3-gallate (EGCG) against porcine reproductive and respiratory syndrome virus infection in vitro. Antiviral Res.

[13] Yang Q, Gao L, Si J, Sun Y, Liu J, Cao L, Feng W (2013). Inhibition of porcine reproductive and respiratory syndrome virus replication by flavaspidic acid AB. Antiviral Res.

[14] Saito T, Owen DM, Jiang F, Marcotrigiano J, Gale M (2008). Innate immunity induced by composition-dependent RIG-I recognition of hepatitis C virus RNA. Nature.

[15] Almeida L, Khare S, Misharin AV, Patel R, Ratsimandresy RA, Wallin MC, Perlman H, Greaves DR, Hoffman HM, Dorfleutner A, Stehlik C (2015). The PYRIN domain-only protein POP1 inhibits inflammasome assembly and ameliorates inflammatory disease. Immunity.

[16] Lama L, Adura C, Xie W, Tomita D, Kamei T, Kuryavyi V, Gogakos T, Steinberg JI, Miller M, Ramos-Espiritu L, Asano Y, Hashizume S, Aida J, Imaeda T, Okamoto R, Jennings AJ, Michino M, Kuroita T, Stamford A, Gao P, Meinke P, Glickman JF, Patel DJ, Tuschl T (2019). Development of human cGAS-specific small-molecule inhibitors for repression of dsDNA-triggered interferon expression. Nat Commun.

[17] Zhao J, Zeng Y, Xu S, Chen J, Shen G, Yu C, Knipe D, Yuan W, Peng J, Xu W, Zhang C, Xia Z, Feng P (2016). A viral deamidase targets the helicase domain of RIG-I to block RNA-induced activation. Cell Host Microbe.

[18] Hotter D, Bosso M, Jønsson KL, Krapp C, Stürzel CM, Das A, Littwitz-Salomon E, Berkhout B, Russ A, Wittmann S, Gramberg T, Zheng Y, Martins LJ, Planelles V, Jakobsen MR, Hahn BH, Dittmer U, Sauter D, Kirchhoff F (2019). IFI16 targets the transcription factor Sp1 to suppress HIV-1 transcription and latency reactivation. Cell Host Microbe.

[19] Gariano GR, Dell'Oste V, Bronzini M, Gatti D, Luganini A, De Andrea M, Gribaudo G, Gariglio M, Landolfo S (2012). The intracellular DNA sensor IFI16 gene acts as restriction factor for human cytomegalovirus replication. PLoS Pathog.

[20] Johnson KE, Bottero V, Flaherty S, Dutta S, Singh VV, Chandran B (2014). IFI16 restricts HSV-1 replication by accumulating on the hsv-1 genome, repressing HSV-1 gene expression, and directly or indirectly modulating histone modifications. PLoS Pathog.

[21] Garcia-Moreno M, Noerenberg M, Ni S, Järvelin AI, González-Almela E, Lenz CE, Bach-Pages M, Cox V, Avolio R, Davis T, Hester S, Sohier TJM, Li B, Heikel G, Michlewski G, Sanz MA, Carrasco L, Ricci EP, Pelechano V, Davis I, Fischer B, Mohammed S, Castello A (2019). System-wide profiling of RNA-binding proteins uncovers key regulators of virus infection. Mol Cell.

[22] Jiang Z, Wei F, Zhang Y, Wang T, Gao W, Yu S, Sun H, Pu J, Sun Y, Wang M, Tong Q, Gao C, Chang KC, Liu J (2021). IFI16 directly senses viral RNA and enhances RIG-I transcription and activation to restrict influenza virus infection. Nat Microbiol.

[23] Chang X, Shi X, Zhang X, Wang L, Li X, Wang A, Deng R, Zhou E, Zhang G (2019). IFI16 inhibits porcine reproductive and respiratory syndrome virus 2 replication in a MAVS-dependent manner in MARC-145 cells. Viruses.

[24] Arend WP, Palmer G, Gabay C (2008). IL-1, IL-18, and IL-33 families of cytokines. Immunol Rev.

[25] Hastie AT, Everts KB, Cho SK, Zangrilli J, Shaver JR, Pollice MB, Fish JE, Peters SP (1996). IL-1 beta release from cultured bronchial epithelial cells and bronchoalveolar lavage cells from allergic and normal humans following segmental challenge with ragweed. Cytokine.

[26] Lanaya H, Natarajan A, Komposch K, Li L, Amberg N, Chen L, Wculek SK, Hammer M, Zenz R, Peck-Radosavljevic M, Sieghart W, Trauner M, Wang H, Sibilia M (2014). EGFR has a tumour-promoting role in liver macrophages during hepatocellular carcinoma formation. Nat Cell Biol.

[27] Kerur N, Veettil MV, Sharma-Walia N, Bottero V, Sadagopan S, Otageri P, Chandran B (2011). IFI16 acts as a nuclear pathogen sensor to induce the inflammasome in response to Kaposi Sarcoma-associated herpesvirus infection. Cell Host Microbe.

[28] Isorce N, Testoni B, Locatelli M, Fresquet J, Rivoire M, Luangsay S, Zoulim F, Durantel D (2016). Antiviral activity of various interferons and pro-inflammatory cytokines in non-transformed cultured hepatocytes infected with hepatitis B virus. Antiviral Res.

[29] Schmitz N, Kurrer M, Bachmann MF, Kopf M (2005). Interleukin-1 is responsible for acute lung immunopathology but increases survival of respiratory influenza virus infection. J Virol.

[30] Li J, Song J, Kang L, Huang L, Zhou S, Hu L, Zheng J, Li C, Zhang X, He X, Zhao D, Bu Z, Weng C (2021). pMGF505-7R determines pathogenicity of African swine fever virus infection by inhibiting IL-1β and type I IFN production. PLoS Pathog.

[31] Shi Y, Li M (2007). Biological effects of toosendanin, a triterpenoid extracted from Chinese traditional medicine. Prog Neurobiol.

[32] Ma Z, Gulia-Nuss M, Zhang X, Brown MR (2013). Effects of the botanical insecticide, Toosendanin, on blood digestion and egg production by female *Aedes aegypti* (diptera: culicidae): topical application and ingestion. J Med Entomol.

[33] Shi YL, Wang ZF (2004). Cure of experimental botulism and antibotulismic effect of toosendanin. Acta Pharmacol Sin.

[34] Cao L, Qu D, Wang H, Zhang S, Jia C, Shi Z, Wang Z, Zhang J, Ma J (2016). Toosendanin exerts an anti-cancer effect in glioblastoma by inducing estrogen receptor beta-and p53-mediated apoptosis. Int J Mol Sci.

[35] Liu X, Wang H, Zhang L, Wang Y, Wang J, Wang P, He X, He Y (2016). Anticancer effects of crude extract from *Melia toosendan* Sieb. et Zucc on hepatocellular carcinoma in vitro and in vivo. Chin J Integr Med.

[36] Wang Q, Wang Z, Hou G, Huang P (2020). Toosendanin suppresses glioma progression property and induces apoptosis by regulating miR-608/Notch axis. Cancer Manag Res.

[37] Jin Y, Kwon S, Choi JG, Cho WK, Lee B, Ma JY (2019). Toosendanin from melia fructus suppresses influenza A virus infection by altering nuclear localization of viral polymerase PA protein. Front Pharmacol.

[38] Watanabe T, Sakamoto N, Nakagawa M, Kakinuma S, Itsui Y, Nishimura-Sakurai Y, Ueyama M, Funaoka Y, Kitazume A, Nitta S, Kiyohashi K, Murakawa M, Azuma S, Tsuchiya K, Oooka S, Watanabe M (2011). Inhibitory effect of a triterpenoid compound, with or without alpha interferon, on hepatitis C virus infection. Antimicrob Agents Chemother.

[39] Li S, Ye M, Chen Y, Zhang Y, Li J, Liu W, Li H, Peng K (2021). Screening of a small molecule compound library identifies Toosendanin as an inhibitor against bunyavirus and SARS-CoV-2. Front Pharmacol.

[40] Brockmeier SL, Loving CL, Eberle KC, Hau SJ, Buckley A, Van Geelen A, Montiel NA, Nicholson T, Lager KM (2017). Interferon alpha inhibits replication of a live-attenuated porcine reproductive and respiratory syndrome virus vaccine preventing development of an adaptive immune response in swine. Vet Microbiol.

[41] Xie J, Zhu W, Chen Y, Wei C, Zhou P, Zhang M, Huang Z, Sun L, Su S, Zhang G (2013). Molecular epidemiology of PRRSV in South China from 2007 to 2011 based on the genetic analysis of ORF5. Microb Pathog.

[42] Greig A (1975). The use of a microtitration technique for the routine assay of African swine fever virus. Arch Virol.

[43] Rao X, Huang X, Zhou Z, Lin X (2013). An improvement of the 2ˆ(-delta delta CT) method for quantitative real-time polymerase chain reaction data analysis, Biostatistics. Biostat Bioinforma Biomath.

[44] Schmittgen TD, Livak KJ (2008). Analyzing real-time PCR data by the comparative C(T) method. Nat Protoc.

[45] Kim Y, Lee C (2013). Ribavirin efficiently suppresses porcine nidovirus replication. Virus Res.

[46] Mansuy-Aubert V, Zhou QL, Xie X, Gong Z, Huang JY, Khan AR, Aubert G, Candelaria K, Thomas S, Shin DJ, Booth S, Baig SM, Bilal A, Hwang D, Zhang H, Lovell-Badge R, Smith SR, Awan FR, Jiang Z (2013). Imbalance between neutrophil elastase and its inhibitor α1-antitrypsin in obesity alters insulin sensitivity, inflammation, and energy expenditure. Cell Metab.

[47] Yang T, Xu R, Huo J, Wang B, Du X, Dai B, Zhu M, Zhan Y, Zhang D, Zhang Y (2021). WWOX activation by toosendanin suppresses hepatocellular carcinoma metastasis through JAK2/Stat3 and Wnt/β-catenin signaling. Cancer Lett.

[48] Zhang S, Cao L, Wang Z, Li Z, Ma J (2019). Anti-cancer effect of toosendanin and its underlying mechanisms. J Asian Nat Prod Res.

[49] Schattgen SA, Fitzgerald KA (2011). The PYHIN protein family as mediators of host defenses. Immunol Rev.

[50] Thompson MR, Sharma S, Atianand M, Jensen SB, Carpenter S, Knipe DM, Fitzgerald KA, Kurt-Jones EA (2014). Interferon γ-inducible protein (IFI) 16 transcriptionally regulates type i interferons and other interferon-stimulated genes and controls the interferon response to both DNA and RNA viruses. J Biol Chem.

[51] Cao L, Ji Y, Zeng L, Liu Q, Zhang Z, Guo S, Guo X, Tong Y, Zhao X, Li C, Chen Y, Guo D (2019). P200 family protein IFI204 negatively regulates type I interferon responses by targeting IRF7 in nucleus. PLoS Pathog.

[52] Wichit S, Hamel R, Yainoy S, Gumpangseth N, Panich S, Phuadraksa T, Saetear P, Monteil A, Morales Vargas R, Missé D (2019). Interferon-inducible protein (IFI) 16 regulates Chikungunya and Zika virus infection in human skin fibroblasts. EXCLI J.

[53] Syed SA, Beurel E, Loewenstein DA, Lowell JA, Craighead WE, Dunlop BW, Mayberg HS, Dhabhar F, Dietrich WD, Keane RW, de Rivero Vaccari JP, Nemeroff CB (2018). Defective inflammatory pathways in never-treated depressed patients are associated with poor treatment response. Neuron.

[54] Koo JE, Shin SW, Um SH, Lee JY (2015). X-shaped DNA potentiates therapeutic efficacy in colitis-associated colon cancer through dual activation of TLR9 and inflammasomes. Mol Cancer.

[55] Jin T, Perry A, Jiang J, Smith P, Curry JA, Unterholzner L, Jiang Z, Horvath G, Rathinam VA, Johnstone RW, Hornung V, Latz E, Bowie AG, Fitzgerald KA, Xiao T (2012). Structures of the HIN domain:DNA complexes reveal ligand binding and activation mechanisms of the AIM2 inflammasome and IFI16 receptor. Immunity.

[56] Garber C, Vasek MJ, Vollmer LL, Sun T, Jiang X, Klein RS (2018). Astrocytes decrease adult neurogenesis during virus-induced memory dysfunction via IL-1. Nat Immunol.

[57] Wang W, Li G, De W, Luo Z, Pan P, Tian M, Wang Y, Xiao F, Li A, Wu K, Liu X, Rao L, Liu F, Liu Y, Wu J (2018). Zika virus infection induces host inflammatory responses by facilitating NLRP3 inflammasome assembly and interleukin-1β secretion. Nat Commun.

[58] Lapuente D, Storcksdieck Genannt Bonsmann M, Maaske A, Stab V, Heinecke V, Watzstedt K, Heß R, Westendorf AM, Bayer W, Ehrhardt C, Tenbusch M (2018). IL-1β as mucosal vaccine adjuvant: the specific induction of tissue-resident memory T cells improves the heterosubtypic immunity against influenza A viruses. Mucosal Immunol.

[59] Ichinohe T, Lee HK, Ogura Y, Flavell R, Iwasaki A (2009). Inflammasome recognition of influenza virus is essential for adaptive immune responses. J Exp Med.

[60] Durrant DM, Robinette ML, Klein R (2013). IL-1R1 is required for dendritic cell-mediated T cell reactivation within the CNS during West Nile virus encephalitis. J Exp Med.

[61] Song Z, Bai J, Nauwynck H, Lin L, Liu X, Yu J, Jiang P (2019). 25-Hydroxycholesterol provides antiviral protection against highly pathogenic porcine reproductive and respiratory syndrome virus in swine. Vet Microbiol.

[62] Aarreberg LD, Wilkins C, Ramos HJ, Green R, Davis MA, Chow K, Gale M (2018). Interleukin-1β signaling in dendritic cells induces antiviral interferon responses. mBio.

[63] Xu J, Sriramula S, Xia H, Moreno-Walton L, Culicchia F, Domenig O, Poglitsch M, Lazartigues E (2017). Clinical relevance and role of neuronal AT(1) receptors in ADAM17-mediated ACE2 shedding in neurogenic hypertension. Circ Res.

[64] Guo L, Niu J, Yu H, Gu W, Li R, Luo X, Huang M, Tian Z, Feng L, Wang Y (2014). Modulation of CD163 expression by metalloprotease ADAM17 regulates porcine reproductive and respiratory syndrome virus entry. J Virol.

[65] Wang X, Wang C, Wang Z (2013). Determination of toosendanin in rat plasma by ultra-performance liquid chromatography-electrospray ionization-mass spectrometry and its application in a pharmacokinetic study. Biomed Chromatogr.

